# A new circular RNA–encoded protein BIRC6-236aa inhibits transmissible gastroenteritis virus (TGEV)–induced mitochondrial dysfunction

**DOI:** 10.1016/j.jbc.2022.102280

**Published:** 2022-07-19

**Authors:** Xiaomin Zhao, Jianxiong Guo, Xinyue Wang, Jiadi Lin, Zhihao Liu, Chunmei Xu, Di Zhang, Dewen Tong

**Affiliations:** College of Veterinary Medicine, Northwest A&F University, Yangling, Shaanxi, P.R. China

**Keywords:** transmissible gastroenteritis virus, mitochondrial permeability transition pore, circBIRC6-2, BIRC6-236aa, voltage-dependent anion-selective channel protein 1 (VDAC1), AM, acetoxymethyl ester, BCF, back-circular frame, CCK8, cell counting kit-8, cDNA, complementary DNA, CypD, Cyclophilin D, DAPI, 4, 6-diamidino-2-phenylindole, FCF, front-circular frame, gDNA, genomic DNA, HA, hemagglutinin, IEM, immunoelectron microscopy, IMM, inner mitochondrial membrane, IP, immunoprecipitation, IPEC-J2, intestinal epithelial cell line jejunum 2, IRES, internal ribosomal entrance site, LR, London Resin, lv, lentiviruse, MMP, mitochondrial membrane potential, MOI, multiplicity of infection, mPTP, mitochondrial permeability transition pore, MS, mass spectrometry, PLA, proximity ligation assay, qRT-PCR, quantitative real-time PCR, Rhod-2, rhodamine-2, TGEV, transmissible gastroenteritis virus

## Abstract

Transmissible gastroenteritis virus (TGEV), a member of the coronavirus family, is the pathogen responsible for transmissible gastroenteritis, which results in mitochondrial dysfunction in host cells. Previously, we identified 123 differentially expressed circular RNAs (cRNA)from the TGEV-infected porcine intestinal epithelial cell line jejunum 2 (IPEC-J2). Previous bioinformatics analysis suggested that, of these, circBIRC6 had the potential to regulate mitochondrial function. Furthermore, mitochondrial permeability transition, a key step in the process of mitochondrial dysfunction, is known to be caused by abnormal opening of mitochondrial permeability transition pores (mPTPs) regulated by the voltage-dependent anion-selective channel protein 1 (VDAC)–Cyclophilin D (CypD) complex. Therefore, in the present study, we investigated the effects of circBIRC6-2 on mitochondrial dysfunction and opening of mPTPs. We found that TGEV infection reduced circBIRC6-2 levels, which in turn reduced mitochondrial calcium (Ca^2+^) levels, the decrease of mitochondrial membrane potential, and opening of mPTPs. In addition, we also identified ORFs and internal ribosomal entrance sites within the circBIRC6-2 RNA. We demonstrate circBIRC6-2 encodes a novel protein, BIRC6-236aa, which we show inhibits TGEV-induced opening of mPTPs during TGEV infection. Mechanistically, we identified an interaction between BIRC6-236aa and VDAC1, suggesting that BIRC6-236aa destabilizes the VDAC1–CypD complex. Taken together, the results suggest that the novel protein BIRC6-236aa encoded by cRNA circBIRC6-2 inhibits mPTP opening and subsequent mitochondrial dysfunction by interacting with VDAC1.

The mitochondrion, an organelle of eukaryotic cells, plays a pivotal role in maintaining normal cellular function (1). Mitochondria are sensitive to various stimuli, including viral infection, leading to potential dysfunction. Dysfunctional mitochondria release signals that induce cell death. Mitochondrial permeability transition pores (mPTPs) are located on the inner mitochondrial membrane (IMM). Normally these pores are closed; prolonged opening of mPTPs results in apoptosis and cell death (2, 3). Persistent opening of mPTPs can occur in response to virus infections or to oxidative or chemical-induced stress (4, 5, 6, 7, 8). Opening of mPTPs increases mitochondrial permeability to molecules with a molecular weight of less than 1.5 kD, as well as mitochondrial permeability transition, resulting in disruption of mitochondrial respiration, swelling and rupture of the mitochondrial membrane, and cell death (9, 10, 11). Transmissible gastroenteritis virus (TGEV) can induce mPTP opening and damage mitochondria (12, 13); however, the underlying mechanism is unclear. Therefore, identifying how TGEV regulates mPTP opening is essential if we are to better understand the pathogenic mechanism of the virus.

Opening of mPTPs is regulated by VDAC1 and Cyclophilin D (CypD) (14, 15). VDAC1 forms an ion channel in the outer mitochondrial membrane and promotes opening of mPTPs (16, 17). CypD, encoded by the *ppif* (peptidyl-prolyl *cis*-*trans* isomerase) gene, serves as a key factor that reverses mPTP opening. It is located in the IMM and interacts with the VDAC1–GRP75–IP3R complex at mitochondria-associated membranes (18, 19). In CypD-knockdown mice, mitochondria are desensitized to Ca^2+^, which is essential for permissiveness to mPTP opening (20, 21, 22). However, the way in which TGEV affects the VDAC1/CypD/mPTP regulatory mechanism remains unknown.

Circular RNAs (cRNAs) are a class of noncoding RNAs implicated in most biological and pathological processes, including mitochondria dysfunction, infectious disease, cell death, and tumorigenesis (23). They play regulatory roles in many ways, including serving as miRNA sponges and protein decoys/dynamic scaffolds, regulating transcription of their encoding genes, interacting with RNA-binding proteins, and encoding proteins (24, 25). Previously, we found that 123 cRNAs were differentially expressed upon TGEV infection (26). CircBIRC6-2 is one such differentially expressed cRNA and encoded by *baculoviral IAP repeat containing 6* (*birc6*) that plays a role in mitochondrial function (27). Bioinformatic analysis suggested that circBIRC6-2 has the ability to adsorb miR-22, whose potential target genes (*e.g.*, hexokinase 2 [HK2], pyruvate dehydrogenase E1 beta subunit [PDHB], and nuclear receptor coactivator 4 [NCOA4]) are related to mitochondria, (12, 17, 26, 28, 29). Therefore, we hypothesized that circBIRC6-2 has the potential to regulate mitochondrial function.

Here, we demonstrate that TGEV downregulates circBIRC6-2, thereby increasing the sensitivity of mitochondrial permeability transition to TGEV. circBIRC6-2 encodes a protein BIRC6-236aa that is located throughout the interior of the mitochondria including mitochondria. In addition, BIRC6-236aa has a suppressive effect on mPTP opening by interacting with VDAC1.

## Results

### TGEV induces mitochondrial damage and opening of mPTPs

**Figure 1:**
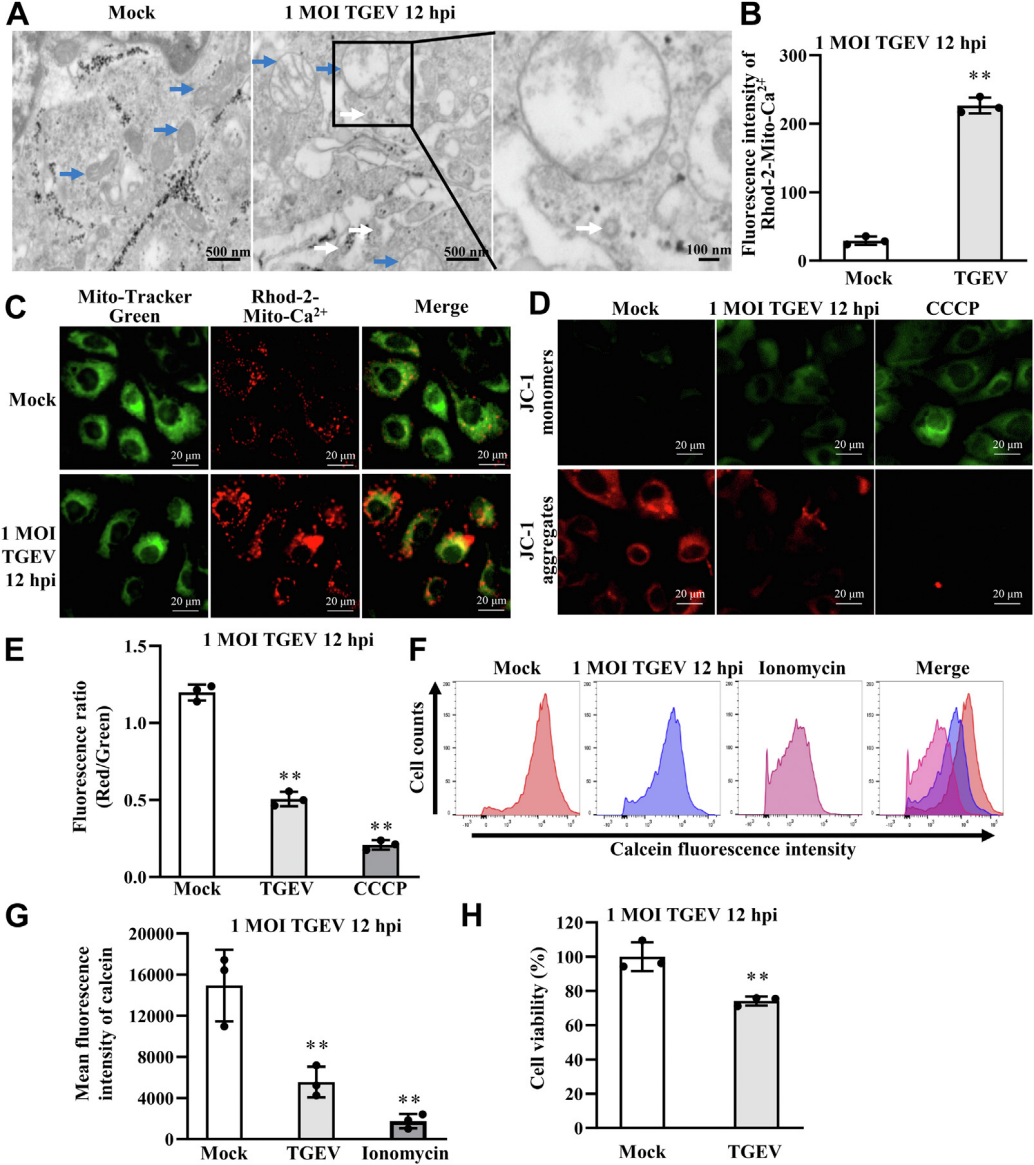
**TGEV induces mitochondrial damage and mPTP opening.***A*, ultrastructural changes in mitochondria in response to TGEV infection. The *blue arrow* points to a mitochondrion. The *white arrow* points to a viral particle. *B*, fold change of mitochondrial Ca^2+^ concentration in response to TGEV infection. Mitochondrial Ca^2+^ concentration upon TGEV infection was detected using Rhod-2 kit which can fluoresce after binding Ca^2+^. The fluorescence intensity was measured with spectrofluorometer. *C*, imaging of Rhod-2 fluorescence was measured with laser scanning confocal microscope after TGEV infection. Green fluorescence indicates mitochondria that are stained with Mito-Tracker Green. Red indicates Ca^2+^ concentration that is stained with Rhod-2. *D*, distribution of JC-1 monomers and JC-1 aggregates in IPEC-J2 cells upon TGEV infection. CCCP, carbonyl cyanide 3-chlorophenylhydrazone, was used as a positive inducer of MMP to promote permeability of the mitochondrial membrane, leading to a reduction in the mitochondrial membrane potential. *Green* indicates JC-1 monomers, and *red* indicates JC-1 aggregates. The increased fluorescence intensity of JC-1 monomers, or decreased fluorescence intensity of JC-1 aggregates, indicates a reduction in the MMP. *E*, the relative fluorescence intensity of *red/green* florescence fell significantly after TGEV infection, indicating that TGEV suppresses the MMP. Ratio of *red/green* fluorescence intensity represents MMP and is positively correlated with MMP. *F* and *G*, TGEV induces mPTP opening in IPEC-J2 cells. The mean *green fluorescence* intensity of calcein indicates the level of mPTP opening. The decrease in the fluorescence intensity of calcein, as measured by flow cytometry, indicates opening of mPTP. Ionomycin was used as a positive control. Ionomycin is a calcium ion carrier that carries massive amounts of extracellular Ca^2+^ into the cell and mitochondrial matrix, thereby contributing to mPTP opening. *H*, variation of cell viability in response to TGEV infection. ∗∗*p* < 0.01, compared with Mock (n = 3). IMM, inner mitochondrial membrane; IPEC-J2, intestinal epithelial cell line jejunum 2; MMP, mitochondrial membrane potential; mPTP, mitochondrial permeability transition pore; TGEV, transmissible gastroenteritis virus.

TGEV infection causes mitochondrial dysfunction (12, 13). Here, we detected morphological changes in the mitochondria of the intestinal epithelial cell line jejunum 2 (IPEC-J2) cells upon infection with TGEV (Fig. 1*A*, *blue arrows*). Histological examination revealed that the mitochondria in infected IPEC-J2 cells had fewer apparent cristae and showed evidence of matrix swelling and rupture of the mitochondrial membrane (Fig. 1*A*, *blue arrows*). TGEV virions were visualized in TGEV-infected cells (Fig. 1*A*, *white arrows*). Studies show that mitochondrial Ca^2+^ overload triggers mitochondrial dysfunction and cell death (30). Therefore, we measured mitochondrial Ca^2+^ concentrations to evaluate mitochondrial damage. To assess mitochondrial Ca^2+^ concentrations, Rhodamine-2 (Rhod-2)-acetoxymethyl ester (AM) (Rhod-2-AM) kit was used. Fluorescence intensity of Ca-Rhod-2 was measured with spectrofluorometer and imaging of Ca-Rhod-2 fluorescence was measured with laser scanning confocal microscope. Fluorescence intensity of Ca-Rhod-2 represents Ca^2+^ concentration. For imaging of Ca-Rhod-2, green fluorescence indicates mitochondria that are stained with Mito-Tracker Green, whereas red fluorescence indicates mitochondrial Ca^2+^ concentration that is stained with Rhod-2. The results revealed that mitochondrial Ca^2+^ concentration was upregulated by TGEV (Fig. 1, *B* and *C*). A reduction of mitochondrial membrane potential (MMP) indicates mitochondrial damage. To assess the variation of MMP after TGEV infection, IPEC-J2 cells were incubated with 5, 5′, 6, 6′-tetrachloro-1, 1′, 3, 3′-tetraethylbenzimidazolocarbocyanine iodide (JC-1) dye, an indicator MMP. JC-1 exhibits potential-dependent accumulation in mitochondria, indicated by a green fluorescence emission (∼529 nm) for the monomeric form of JC-1, which shifts to red (∼590 nm) with a concentration-dependent formation of red fluorescent aggregates of JC-1. Consequently, mitochondrial depolarization is indicated by a decrease in the red/green fluorescence intensity ratio. Thus, the ratio of red/green fluorescence intensity represents MMP and is positively correlated with MMP. Carbonyl cyanide 3-chlorophenylhydrazone is an inducer of MMP, so it is used as the positive control. We observed an increase in the fluorescence intensity of JC-1 monomers and a decrease in the fluorescence intensity of JC-1 aggregates, suggesting a decrease in the MMP (Fig. 1*D*). TGEV infection reduced the red/green signal ratio, suggesting that TGEV reduces the MMP (Fig. 1*E*), leading to mitochondrial damage, in IPEC-J2 cells. Next, we used a calcein-AM to assess opening of mPTPs. The fluorescence intensity of the calcein, as measured by flow cytometry, fell significantly after TGEV infection (Fig. 1, *F* and *G*), suggesting that TGEV triggers opening of mPTPs, leading to mitochondrial damage. In order to assess the effect of calcein on viability, we detected the change of viability upon to calcein using cell counting kit-8 (CCK8). The results showed that calcein has no significant influence on cell viability (Fig. S1). We also examined the effect of TGEV-induced mPTP opening on cell viability using CCK8, and the results showed that TGEV-induced mPTP opening reduced cell viability (Fig. 1*H*).

### CircBIRC6-2 in IPEC-J2 cells is downregulated by TGEV

**Figure 2:**
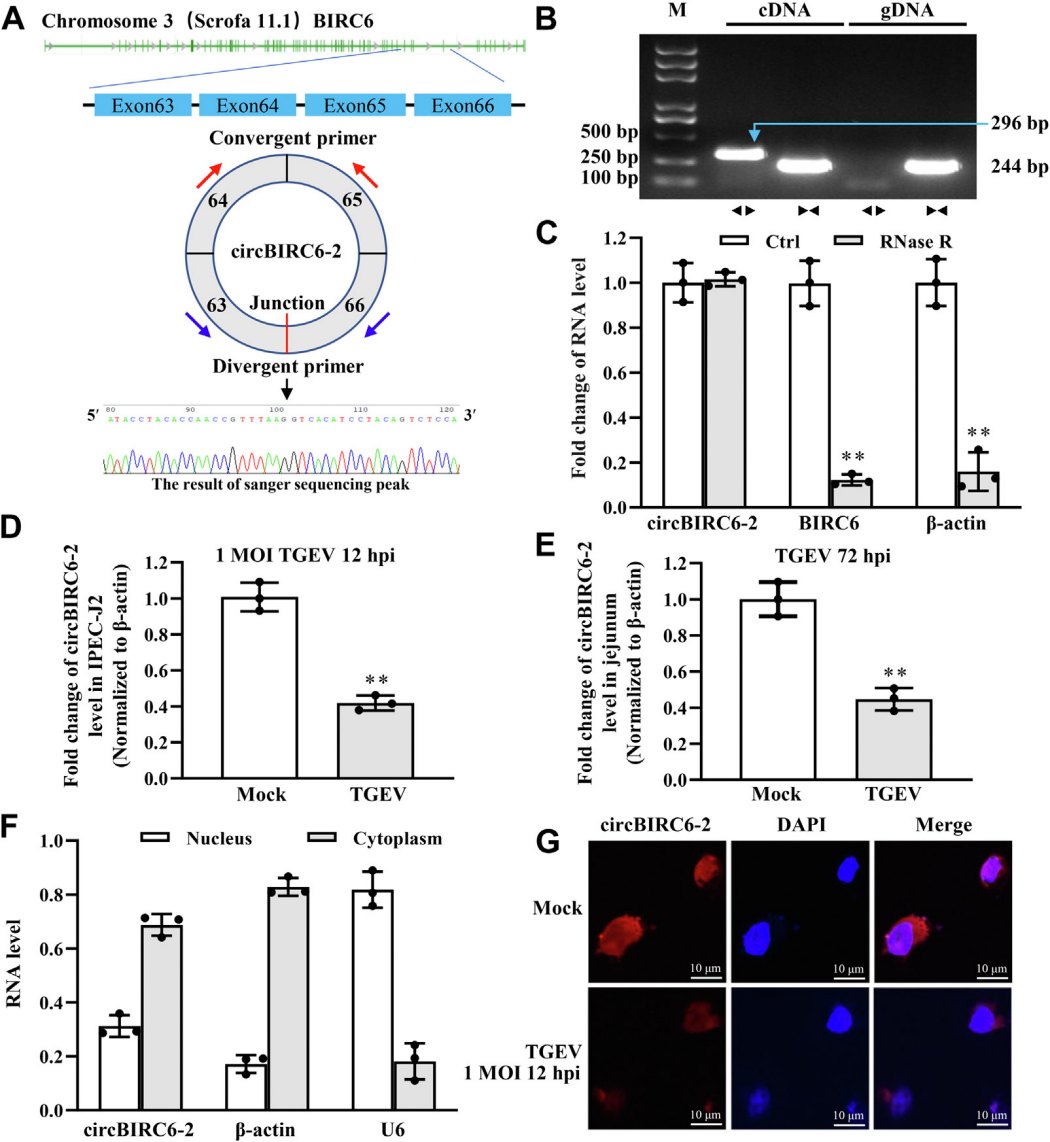
**CircBIRC6-2 in IPEC-J2 cells is downregulated by TGEV.***A*, chromosome localization and splicing of circBIRC6-2. CircBIRC6-2 comprises Exons 63 to 66 of the parental gene BIRC6. *Red* and *blue arrows* represent the amplification direction of the convergent and divergent primers, respectively. The *lower panel* shows the sequence of the junction region within circBIRC6-2. *B*, verification of circBIRC6-2 circular RNA (cRNA) by PCR. gDNA and cDNA were amplified by PCR using convergent and divergent primers to confirm the cRNA conformation. **►◂**: amplification direction of convergent primers. **◂►**: amplification direction of divergent primers. *C*, verification of cRNA circBIRC6-2 using exonuclease RNase R and qRT-PCR. Total RNA from IPEC-J2 cells was treated with exonuclease RNase R and qRT-PCR to confirm the presence of circular RNA. *D* and *E*, TGEV inhibits circBIRC6-2 in both IPEC-J2 cells and porcine jejunum tissue. *F* and *G*, circBIRC6-2 localizes to the cytoplasm and nucleus. RNA extracted from cytoplasmic and nuclear fractions was amplified by qRT-PCR (*F*). RNA FISH was used to detect circBIRC6-2 (*G*). *Red fluorescence* denotes circBIRC6-2, and *blue fluorescence* denotes nuclei. ∗∗*p* < 0.01, compared with the control or Mock (n = 3). cDNA, complementary DNA; gDNA, genomic DNA; IPEC-J2, intestinal epithelial cell line jejunum 2; qRT-PCR, quantitative real-time PCR; TGEV, transmissible gastroenteritis virus.

Previously, we reported that circ5884 is downregulated upon TGEV infection (12, 26). Circ5884 is derived from exon 63, exon 64, exon 65, and exon 66 of the *birc6* gene (Gene ID: 100049694) on chromosome 3 (Scrofa11.1) by back splicing of exon 63 and exon 66. Exons 2 to 9 of the *birc6* gene encode circBIRC6 (31); therefore, we renamed circ5884 as circBIRC6-2 (Fig. 2*A*).

Next, to verify whether exons 63 to 66 of *birc6* can form a cRNA, we used convergent and divergent primers to amplify the linear form of the *birc6* gene and then back spliced the transcripts (Fig. 2*A*). We then used Sanger sequencing to confirm the splice junction within circBIRC6-2 (Fig. 2*A*). Convergent and divergent primers were also used to amplify genomic DNA (gDNA) and complementary DNA (cDNA). A predicted DNA fragment was obtained from cDNA by PCR using divergent primers, but no amplicon was generated from gDNA (Fig. 2*B*). Unlike linear RNA, circBIRC6-2 was not degraded by exonuclease RNase R (Fig. 2*C*), indicating that circBIRC6-2 is circular.

Next, we measured circBIRC6-2 levels in IPEC-J2 cells and porcine jejunum tissues with or without TGEV-infected by quantitative real-time PCR (qRT-PCR); the results show that circBIRC6-2 was downregulated in both (Fig. 2, *D* and *E*). Nucleus/cytoplasm fractionation and RNA FISH were then performed to ascertain the subcellular localization of circBIRC6-2. The results showed that circBIRC6-2 localized in both the nucleus and the cytoplasm (Fig. 2, *F* and *G*).

### CircBIRC6-2 inhibits TGEV-induced opening of mPTPs

**Figure 3:**
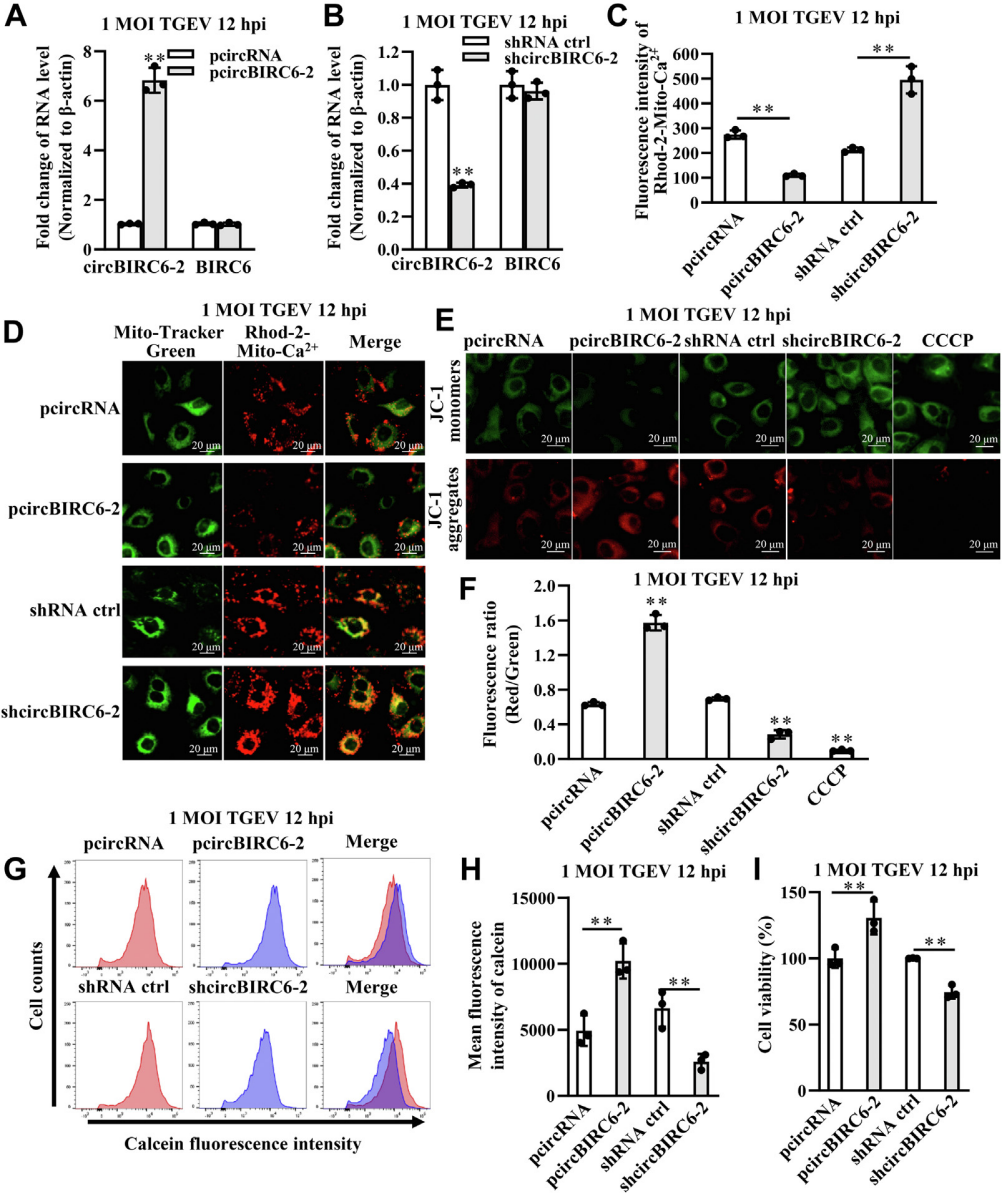
**CircBIRC6-2 inhibits TGEV-induced opening of mPTP.***A*, circBIRC6-2 overexpressed from pcircBIRC6-2 had no significant effect on expression of the parental gene BIRC6. *B*, circBIRC6-2 was silenced by shcircBIRC6-2; however, shcircBIRC6-2 had no significant effect on BIRC6 at the mRNA level. *C*, mitochondrial Ca^2+^ concentrations, as measured by spectrofluorometer, fell upon overexpression of circBIRC6-2 and increased upon silencing of circBIRC6-2, after TGEV infection at 1 MOI for 12 h. *D*, variation of Rhod-2 fluorescence, as measured by laser scanning confocal microscope. *E*, circBIRC6-2 prevented reduction in the MMP. The fluorescence intensity of JC-1 monomers decreased and that of JC-1 aggregates increased, upon overexpression of circBIRC6-2 after TGEV infection at 1 MOI for 12 h, whereas that of JC-1 monomers increased and that of JC-1 aggregates decreased, upon silencing of circBIRC6-2. *F*, the relative *red/green* fluorescence intensity increased upon overexpression of circBIRC6-2 and decreased upon silencing of circBIRC6-2, after TGEV infection at 1 MOI for 12 h. *G* and *H*, the mean fluorescence intensity of calcein, as measured by flow cytometry, increased upon overexpression of circBIRC6-2 and decreased upon silencing of circBIRC6-2 after TGEV infection at 1 MOI for 12 h. *I*, variation of cell viability upon overexpression of circBIRC6-2 and decreased upon silencing of circBIRC6-2 after TGEV infection at 1 MOI for 12 h. ∗∗*p* < 0.01, compared with the control group (n = 3). MOI, multiplicity of infection; mPTP, mitochondrial permeability transition pore; TGEV, transmissible gastroenteritis virus.

TGEV downregulates circBIRC6-2 and promotes opening of mPTPs; therefore, we speculated that circBIRC6-2 might play a role in this process. To investigate the effects of circBIRC6-2 on mPTP opening, we constructed recombinant plasmids pcircBIRC6-2 (overexpressing circBIRC6-2) and shcircBIRC6-2 (producing a shRNA targeting circBIRC6-2); these plasmids led to marked overexpression or repression, respectively, of circBIRC6-2 (Fig. 3, *A* and *B*). Mitochondrial Ca^2+^ concentrations in TGEV-infected cells was measured by spectrofluorometer and fell significantly in the presence of pcircBIRC6-2 and increased in the presence of shcircBIRC6-2 with TGEV infection at 1 multiplicity of infection (MOI) for 12 h (Fig. 3*C*). Changes in red fluorescence intensity were observed using laser scanning confocal microscope and suggested a similar change in Ca^2+^ concentrations (Fig. 3*D*). In addition, pcircBIRC6-2 reduced the fluorescence intensity of JC-1 monomers and increased that of JC-1 aggregates with TGEV infection at 1 MOI for 12 h. The opposite occurred in the presence of shcircBIRC6-2. These results indicate that circBIRC6-2 inhibits the TGEV-induced reduction in the MMP (Fig. 3, *E* and *F*). With TGEV infection at 1 MOI for 12 h, the mean fluorescence intensity of calcein, as measured by flow cytometry, increased in the presence of pcircBIRC6-2 and fell in the presence of shcircBIRC6-2 (Fig. 3, *G* and *H*). Finally, pcircBIRC6-2 increased cell viability, whereas shcircBIRC6-2 decreased it (Fig. 3*I*). Taken together, the aforementioned results suggest that circBIRC6-2 suppresses TGEV-induced opening of mPTPs.

### CircBIRC6-2 encodes the protein BIRC6-236aa

**Figure 4:**
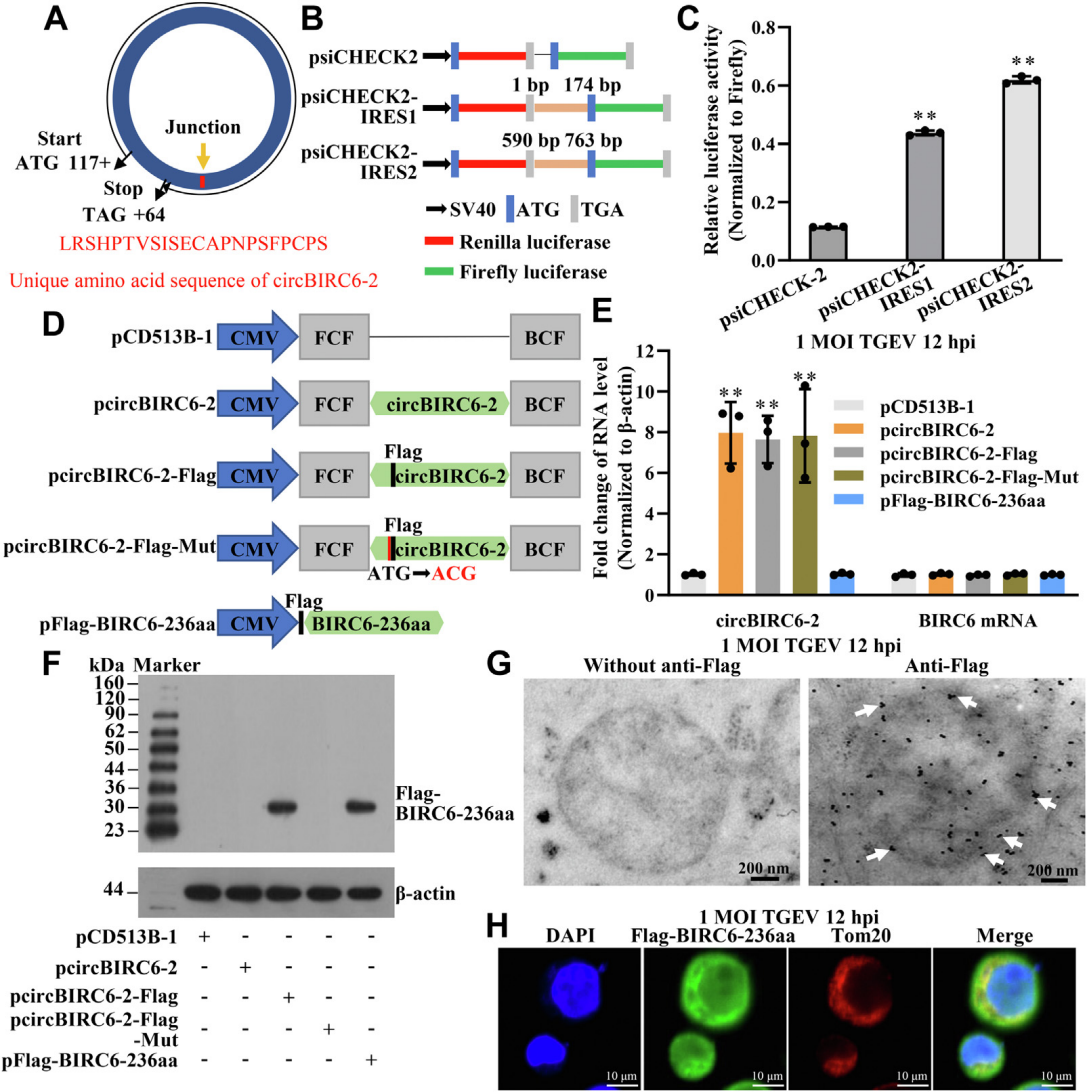
**CircBIRC6-2 encodes the protein BIRC6-236aa.***A*, illustration of the circBIRC6-2-encoded protein. *B*, the IRES sequences of circBIRC6-2 were cloned into psiCHECK2. *C*, putative IRES activity was tested in a dual-luciferase reporter assay. *D*, illustration outlining plasmid construction. Recombinant plasmid pcircBIRC6-2: the circBIRC6-2 sequence was cloned into the pCD513B-1 vector. The FCF (front-circular frame) and BCF (back-circular frame) were added to the 5′ and 3′ ends, respectively, of the circBIRC6-2 sequence to guarantee production of cRNA. Recombinant plasmid pcircBIRC6-2-Flag: a Flag-tag sequence was inserted after the “ATG” initiation codon in pcircBIRC6-2. Recombinant plasmid pcircBIRC6-2-Flag-Mut: the initiation codon “ATG” of BIRC6-236aa was mutated to “ACG” in the pcircBIRC6-2-Flag vector; therefore, it does not translate a protein. Recombinant plasmid pFlag-BIRC6-236aa: this plasmid generates linear RNAs encoding BIRC6-236aa. *E*, relative expression of circBIRC6-2 and BIRC6 RNA in IPEC-J2 cells transfected with four recombinant plasmids. *F*, analysis of BIRC6-236aa protein in IPEC-J2 cells transfected with the four recombinant plasmids. *G*, subcellular localization of BIRC6-236aa, as demonstrated by IEM. The proteins pointed by *white arrows* were located in mitochondrial outer membrane. *H*, IF was performed to identify localization of BIRC6-236aa in mitochondria. IF result showed that BIRC6-236aa interacts with Tom20. ∗∗*p* < 0.01 in comparison with the control group (n = 3). IEM, immunoelectron microscopy; IF, immunofluorescence; IPEC-J2, intestinal epithelial cell line jejunum 2; IRES, internal ribosomal entrance site.

Recent studies demonstrate that cRNA likely encodes a protein. Because circBIRC6-2 localizes to the nucleus and cytoplasm; it is also likely to encode a protein. Therefore, we examined potential ORFs of circBIRC6-2 using ORF Finder (https://www.ncbi.nlm.nih.gov/orffinder/). A conserved ORF was identified in the circBIRC6-2 sequence that had the potential to encode a protein of 236 aa; we named this putative protein BIRC6-236aa (Fig. 4*A*). To encode a protein, a cRNA must contain an internal ribosomal entrance site (IRES), which is required for initiation of translation in a 5′-cap-independent manner. Therefore, we cloned two potential IRES sequences of circBIRC6-2, IRES1 (1–174 bp), and IRES2 (590–763 bp), into the dual-luciferase reporter vector psiCHECK2 (Fig. 4*B*). The dual-luciferase assay results showed that both IRES1 and IRES2 acted as IRES-like sequences (Fig. 4*C*).

**Figure 5:**
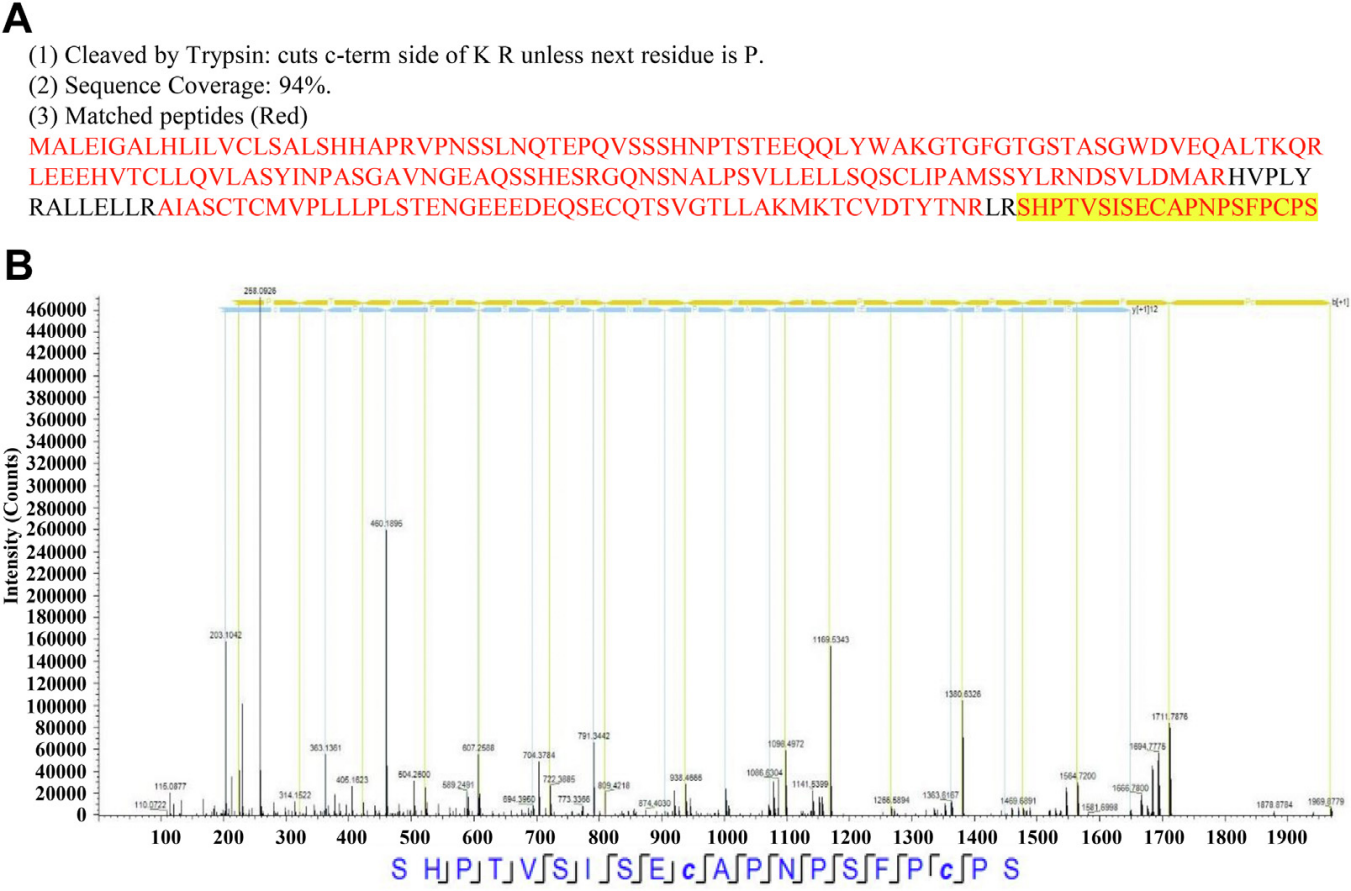
**Identification of BIRC6-236aa using LC-MS/MS.***A*, LC-MS/MS was used to identify the BIRC6-236aa amino acid sequence encoded by circBIRC6-2. The unique amino acid sequence within BIRC6-236aa is marked in *yellow*. 94% amino acid sequence of BIRC6-236aa was identified by LC-MS/MS. *B*, amino acid chromatogram of the unique amino acid sequence within BIRC6-236aa.

To confirm expression of the protein BIRC6-236aa, we constructed four recombinant plasmids for use in packaging lentiviruses (lvs): pcircBIRC6-2, pcircBIRC6-2-Flag, pcircBIRC6-2-Flag-Mut, and pFlag-BIRC6-236aa (Fig. 4*D*). Next, IPEC-J2 cells were infected with lv. We found that circBIRC6-2 was overexpressed by pcircBIRC6-2, pcircBIRC6-2-Flag, and pcircBIRC6-2-Flag-Mut (Fig. 4*E*). Western blot analysis of IPEC-J2^lv-circBIRC6-2-Flag^ and IPEC-J2^lv-Flag-BIRC6-236aa^ cell lysates revealed a band of about 30 kDa (Fig. 4*F*). Because circBIRC6-2 inhibits mPTP opening, we speculated that BIRC6-236aa (encoded by *circBIFC6-2*) might localize to the mitochondria. Indeed, immunoelectron microscopy (IEM) confirmed that BIRC6-236aa was located in cytoplasm, mitochondria matrix, and mitochondrial outer membrane (pointed by *white arrows*) (Fig. 4*G*). Immunofluorescence analysis revealed that BIRC6-236aa was present in both the cytoplasm and the nucleus and that it also interacted with translocase of the outer membrane 20 (Tom20), which is a mitochondrial marker protein in the cytoplasm (Fig. 4*H*). Next, we used LC-MS/MS to identify BIRC6-236aa. About 94% amino acid sequence of BIRC6-236aa was identified by LC-MS/MS. About 1 to 216 aa of BIRC6-236aa was identical to the BIRC6, which was the parental gene of circBIRC6-2 (Fig. 5*A*). In addition, 217 to 236 aa (SHPTVSISECAPNPSFPCPS) was a unique sequence to BIRC6-236aa (Fig. 5*B*). Thus, circBIRC6-2 encodes a protein named BIRC6-236aa.

We predicted the mitochondrial targeting sequence of BIRC6-236aa using MitoProt (https://ihg.helmholtz-muenchen.de/ihg/mitoprot.html) (32), iMLP (http://imlp.bio.uni-kl.de/) (33), and PSORT II (https://psort.hgc.jp/form2.html). The prediction results show that BIRC6-236aa has a potential for targeting mitochondria (Fig. S2).

We analyzed the features of BIRC6-236aa, including hydrophilicity, transmembrane domain, signal peptide, biological function, and advanced structure. The basic physicochemical properties of BIRC6-236aa protein were predicted using the online software ProtParam (https://web.expasy.org/protparam/) (34). The results showed that the BIRC6-236aa protein is composed of 236 amino acids with the molecular formula of C_1097_H_1762_N_308_O_360_S_14_ and contains 24 negatively charged and 13 positively charged amino acid residues with a theoretical pI of 5.10. The theoretical instability coefficient is 60.09 (Fig. S3*A*. BIRC6-236aa is hydrophilic (Fig. S3*B* and has no transmembrane region Fig. S3*C* and signal peptide region with amino acids 1 to 17 as signal peptide positions (Fig. S3*D*. The biological function prediction of BIRC6-236aa protein using InterProScan online software (http://www.ebi.ac.uk/interpro/) showed that BIRC6-236aa protein function is unknown with a segment of signal peptide sequence (Fig. S3*E*. Sopmai (https://prabi.ibcp.fr/htm/site/web/home) and iTASSER (https://zhanggroup.org//I-TASSER/) online softwares were used to analyze the secondary and tertiary structure of BIRC6-236aa. The protein secondary structure prediction results showed that 108 (45.76%) α-helices, 9 (3.81%) β-turns, 10 (4.24%) β-folds, and 109 (46.19%) random coils were predicted (Fig. S3*F*. The results of tertiary structure prediction showed that the 3D spatial structure of BIRC6-236aa protein had more α-helices with irregular coiling (Fig. S3*G*, which was basically consistent with the results of secondary structure prediction.

### BIRC6-236aa downregulates TGEV-induced opening of mPTP opening

**Figure 6:**
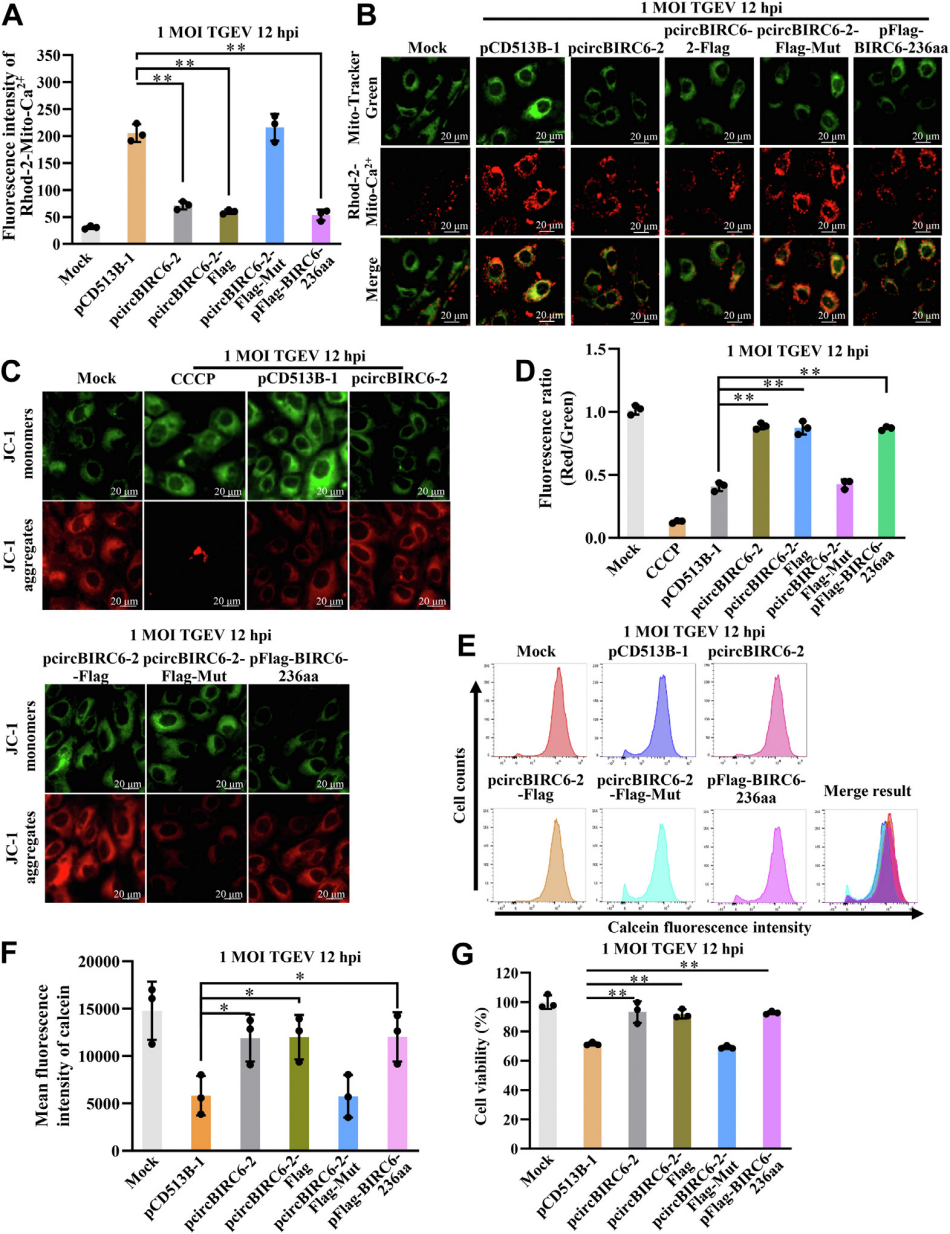
**BIRC6-236aa downregulates TGEV-induced mPTP opening.***A* and *B*, BIRC6-236aa downregulates mitochondrial Ca^2+^ concentration, as measured by spectrofluorometer and laser scanning confocal microscope. *C*, and *D*, BIRC6-236aa inhibits the TGEV-induced decrease in the MMP. Distribution of JC-1 monomers and JC-1 aggregates in IPEC-J2 cells overexpressing the four recombinant plasmids. *E* and *F*, the mean fluorescence intensity of calcein, as measured by flow cytometry, increased upon overexpression of BIRC6-236aa. *G*, CCK8 results showed that the cell viability increased upon overexpression of BIRC6-236aa after TGEV infection at 1 MOI for 12 h. ∗∗*p* < 0.01, compared with the empty vector group (n = 3). CCK8, cell counting kit 8; MOI, multiplicity of infection; MMP, mitochondrial membrane potential; mPTP, mitochondrial permeability transition pore; TGEV, transmissible gastroenteritis virus.

Next, we investigated the effects of BIRC6-236aa on mitochondrial Ca^2+^ concentrations, MMP, and mPTPs. Rhod-2-AM kit was used to detect mitochondrial Ca^2+^ concentration. Mitochondrial Ca^2+^ concentrations in IPEC-J2^lv-circBIRC6-2^, IPEC-J2^lv-circBIRC6-2-Flag^, and IPEC-J2^lv-Flag-BIRC6-236aa^ cells, as measured by spectrofluorometer, were lower than those in control pCD513B-1 cells (Fig. 6*A*); the red fluorescence intensity in IPEC-J2^lv-circBIRC6-2^, IPEC-J2^lv-circBIRC6-2-Flag^, and IPEC-J2^lv-Flag-BIRC6-236aa^ cells, as measured by laser scanning confocal microscope, have the same results (Fig. 6*B*). JC-1 kit was used to detect MMP. The fluorescence intensity generated by JC-1 monomers decreased, while those of JC-1 aggregates increased, in IPEC-J2^lv-circBIRC6-2^, IPEC-J2^lv-circBIRC6-2-Flag^, and IPEC-J2^lv-Flag-BIRC6-236aa^ cells compared with pCD513B-1 cells after TGEV infection (Fig. 6, *C* and *D*), indicating that BIRC6-236aa inhibits the decrease in MMP induced by TGEV. In addition, the mean fluorescence intensity of the calcein, as measured by flow cytometry, increased in IPEC-J2^lv-circBIRC6-2^, IPEC-J2^lv-circBIRC6-2-Flag^, and IPEC-J2^lv-Flag-BIRC6-236aa^ cells (Fig. 6, *E* and *F*), as did cell viability (Fig. 6*G*). Mitochondrial Ca^2+^ concentrations, MMP, opening of mPTP level, and cell viability were not significantly different between IPEC-J2^lv-circBIRC6-2-Mut^ cells and control cells. Taken together, these findings suggest that BIRC6-236aa inhibits mPTP opening.

### BIRC6-236aa interacts with VDAC1

**Figure 7:**
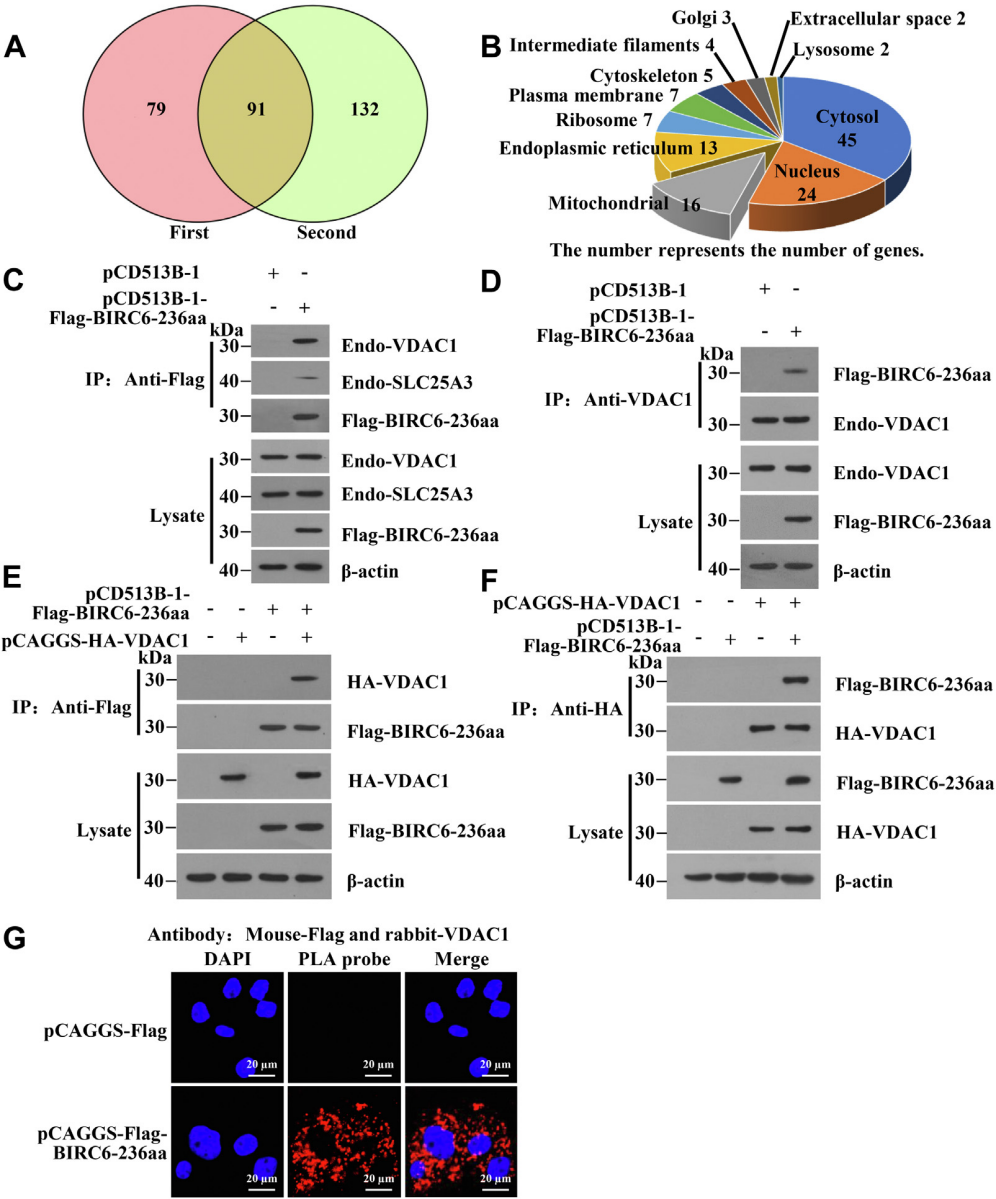
**BIRC6-236aa interacts with VDAC1.***A*, proteins interacting with BIRC6-236aa. In total, LC-MS/MS identified 91 proteins interacting with BIRC6-236aa in two independent replicates (n = 2). *B*, subcellular localization of the interacting proteins. *C*, Flag-BIRC6-236aa interacts with endo-VDAC1. Flag-BIRC6-236aa is overexpressed in IPEC-J2 cells. The interaction between Flag-BIRC6-236aa and endo-VDAC1 or endo-SLC25A3 was identified in an IP assay. *D*, endo-VDAC1 interacts with Flag-BIRC6-236aa. Flag-BIRC6-236aa is overexpressed in IPEC-J2 cells. The interaction between endo-VDAC1 and Flag-BIRC6-236aa was identified in an IP assay. *E* and *F*, Flag-BIRC6-236aa interacts with HA-VDAC1. Flag-BIRC6-236aa and HA-VDAC1 are overexpressed in IPEC-J2 cells. The interaction between Flag-BIRC6-236aa and HA-VDAC1 was identified in an co-IP assay. *G*, a PLA was conducted to detect the interaction between BIRC6-236aa and VDAC1. Mouse anti-Flag and rabbit anti-VDAC1 were used as primary antibodies. Oligonucleotide-linked antimouse IgG and anti-rabbit IgG were used as second antibodies. The pCAGGS-Flag empty vector was used as a negative control. HA, hemagglutinin; IP, immunoprecipitation; IPEC-J2, intestinal epithelial cell line jejunum 2; PLA, proximity ligation assay.

IPEC-J2^lv-circBIRC6-2-Flag^ cells were infected with TGEV for 12 h, followed by immunoprecipitation(IP)-mass spectrometry (MS) to analyze proteins that interact with BIRC6-236aa in two independent replicates. About 170 and 223 potential proteins excluding IgG groups were respectively obtained. Take the intersection of the two experiment, we identified 91 proteins interacted with BIRC6-236aa (Fig. 7*A* and Table S1). Subcellular localization analysis (Fig. 7*B*), Kyoto encyclopedia of genes and genomes (KEGG) analysis (Fig. S4) and gene ontology term analysis (Fig. S5) showed that 16 proteins were located in the mitochondria. Proteins enriched in mitochondria were related mainly to biological process such as cysteine and methionine metabolism; carbon metabolism; and alanine, aspartate, and glutamate metabolism. The results suggest that BIRC6-236aa plays a role in mPTP opening by interacting with host proteins.

VDAC1 and solute carrier family 25 member 3 (SLC25A3) display channel activity during mPTP opening (35), and both have the potential to interact with BIRC6-236aa. To confirm this, we performed an IP assay with Flag-BIRC6-236aa. The results showed that Flag-BIRC6-236aa interacted with endogenous VDAC1 (endo-VDAC1) and SLC25A3 (endo-SLC25A3) (Fig. 7, *C* and *D*), although the interaction with VDAC1 was much stronger. Next, Flag-BIRC6-236aa and hemagglutinin (HA)-VDAC1 were coexpressed in IPEC-J2 cells, and the interaction between them was confirmed by co-IP (Fig. 7, *E* and *F*). Next, we used a DuoLink proximity ligation assay (PLA) to analyze protein–protein interactions (36). Anti-Flag and anti-VDAC1 were used as primary antibodies in the PLA. A strong red signal was observed in IPEC-J2 cells transfected with pCAGGS-Flag-BIRC6-236aa (Fig. 7*G*), suggesting that BIRC6-236aa interacts with VDAC1. Taken together, the data suggest that BIRC6-236aa interacts with VDAC1 to form a complex in IPEC-J2 cells.

### BIRC6-236aa destabilizes the interaction between VDAC1 and CypD

**Figure 8:**
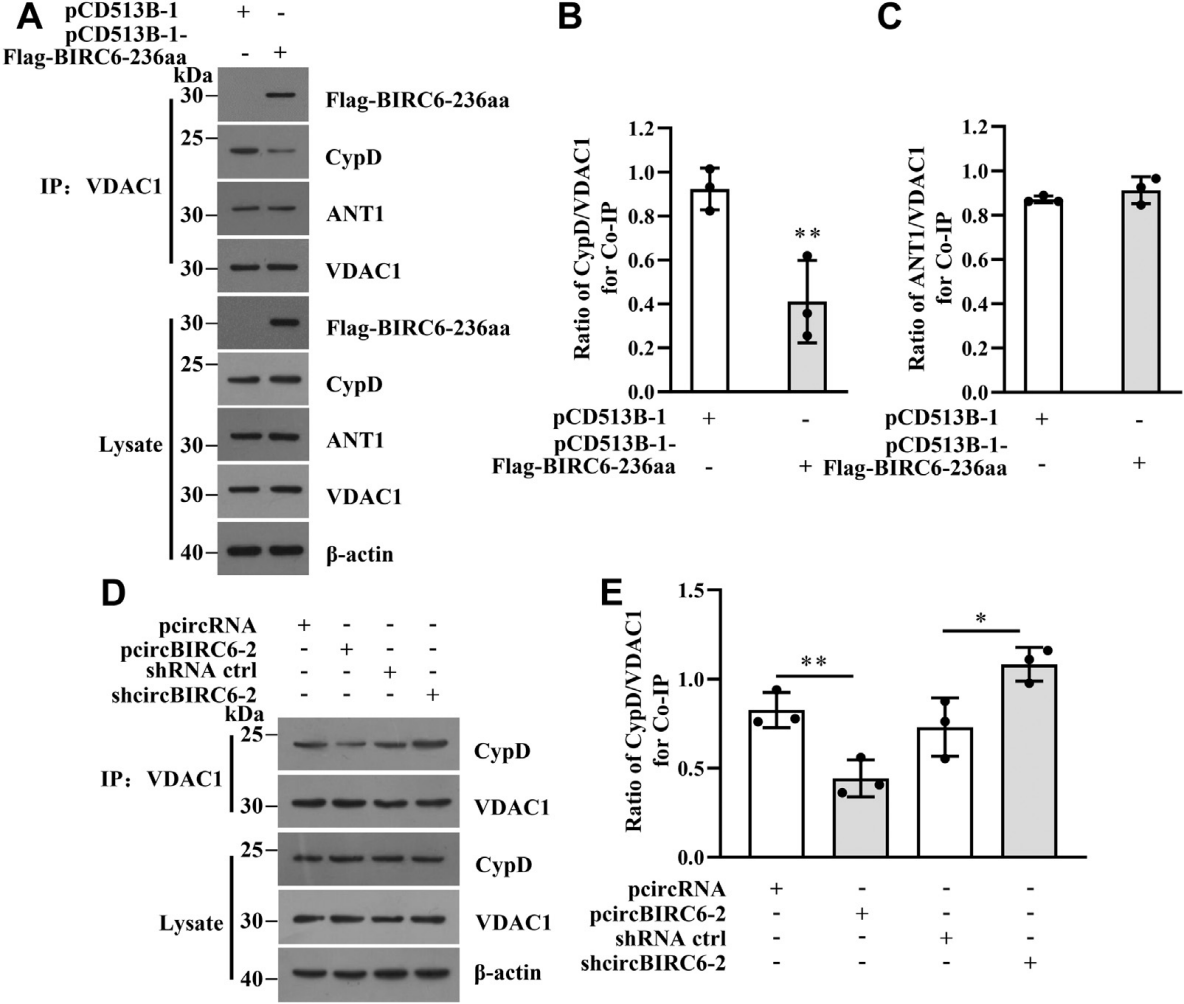
**BIRC6-236aa destabilizes the interaction between VDAC1 and CypD.***A*–*C*, BIRC6-236aa attenuates the interaction between VDAC1 and CypD. The interaction between VDAC1 and CypD or ANT1 was identified in a co-IP assay. BIRC6-236aa does not affect the interaction between VDAC1 and ANT1. Flag-BIRC6-236aa is overexpressed in IPEC-J2 cells. *D* and *E*, circBIRC6-2 weakens the interaction between VDAC1 and CypD. The interaction between VDAC1 and CypD was confirmed in a co-IP assay after overexpression and knockdown of circBIRC6-2, respectively. ∗∗*p* < 0.01, compared with the control (n = 3). CypD, Cyclophilin D; IP, immunoprecipitation.

A complex comprising VDAC1, CypD, and ADP/ATP translocase 1 (ANT1) is essential for mPTP opening (16). Because BIRC6-236aa interacts with VDAC1, we next tested the effect of BIRC6-236aa on the formation of this complex. An IP assay using an anti-VDAC1 antibody revealed that although VDAC1, CypD, and ANT1 formed a complex, the CypD signal was weaker in the presence of BIRC6-236aa, indicating that overexpression of BIRC6-236aa weakens the interaction between VDAC1 and CypD (Fig. 8, *A*–*C*). This suggests that BIRC6-236aa affects the interaction between VDAC1 and CypD but not that between VDAC1 and ANT1. The interaction between VDAC1 and CypD was weakened by overexpression of circBIRC6-2 and strengthened by inhibition of circBIRC6-2 (Fig. 8, *D* and *E*).

**Figure 9:**
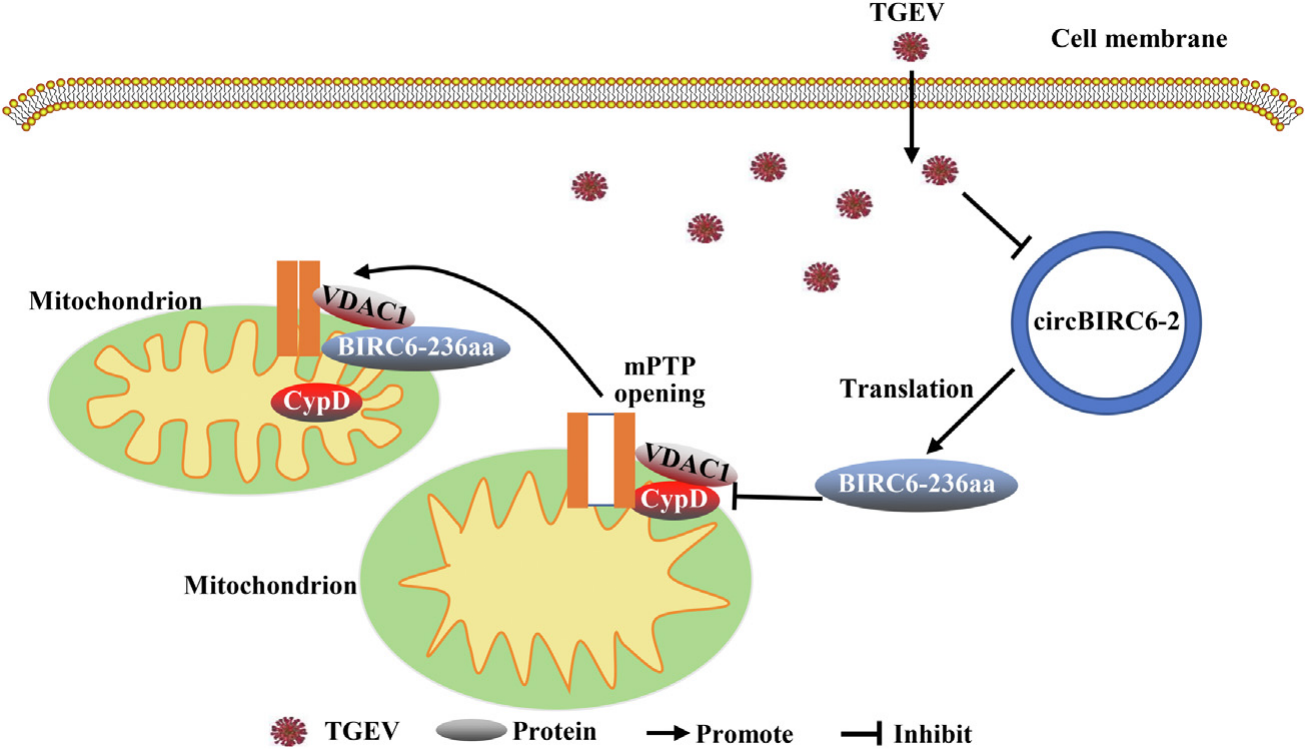
**Illustration of the mechanism underlying circBIRC6-2-regulated mPTP opening.** mPTP, mitochondrial permeability transition pore.

Overall, these data show that circBIRC6-2 encodes a protein, BIRC6-236aa, that destabilizes the interaction between VDAC1 and CypD, thereby inhibiting mPTP opening (Fig. 9).

## Discussion

Previously, we showed that TGEV induces cell death *via* FasL- and mitochondria-mediated pathways (37). The mPTP, a pore in the IMM, is thought to be a key contributor to mitochondrial damage and cell death. Previously, we reported that expression of circBIRC6-2 is reduced by TGEV infection; therefore, we speculated that circBIRC6-2 plays a role in TGEV-induced mitochondrial damage by regulating mPTPs. The findings presented herein demonstrate that circBIRC6-2 inhibits TGEV-induced opening of mPTPs.

In this study, TGEV can induce mPTP opening. It has been reported that mPTP opening leads to cell death, especially necrosis, which can reduce cell viability (38, 39). Here, we found that TGEV induced mPTP opening and decreased cell viability, suggesting that mPTP opening could reduce cell viability in the experimental model. In addition, BIRC6-236aa could increase the cell viability by inhibiting mPTP opening. However, TGEV-induced mPTP opening leads to cell necrosis and the relationship between TGEV-induced cells necrosis and cell viability need to be verified by experiments.

cRNAs are a class of circular RNA molecule that exerts various biological and pathological functions by acting as miRNA sponges, binding to proteins, and encoding polypeptides (40, 41, 42, 43, 44, 45, 46). For example, circ-SHPRH suppresses tumorigenesis of glioma cells by encoding the protein SHPRH (47), and the novel protein circFAM188B-103aa, encoded by circFAM188B promotes proliferation, but inhibits differentiation, of chicken skeletal muscle satellite cells (48). Because a cRNA has no 5′ and 3′ polarity, a ribosome entry sequence is required to initiate translation (49, 50). Here, we found that circBIRC6-2 contains an ORF and two IRES-like sequences. Furthermore, circBIRC6-2 encodes a protein, BIRC6-236aa, which inhibits mPTP opening. These data provide new insight into the mechanism underlying TGEV-induced mitochondrial damage.

The IEM results showed that BIRC6-236aa is distributed not only in the mitochondrial outer membrane but also in the mitochondria matrix and cytoplasm (Fig. 4*G*). Because VDAC1 is located in the mitochondrial outer membrane (51), our results provide evidence that BIRC6-236aa interacts with VDAC1. IP-MS identified 91 proteins that interact with BIRC6-236aa. Subcellular localization analysis revealed that 16 of these proteins are located in mitochondria and 45 proteins are located in the cytosol. Some, such as GOT2, GLUD1, and MDH2, are located in the mitochondrial matrix, suggesting that BIRC6-236aa interacts with mitochondrial proteins in the mitochondrial matrix. Thus, BIRC6-236aa regulates mitochondrial function by interacting with other proteins.

mPTP, a channel of the mitochondrial F1F0-ATP synthase C-subunit, is regulated by VDAC1, CypD, SLC25A3, ANT1, HK2, translocator protein (TSPO), and casein kinase 2 (CK2) (52, 53). The C-subunit of F1F0-ATP synthase forms the framework of the mPTP, whereas regulatory components control opening of the mPTP by regulating protein expression or structure.

VDAC1 and CypD are indispensable components that regulate opening of mPTPs (16, 20). The interaction between VDAC1 and CypD is crucial for formation of the VDAC1–CypD–ANT1 complex required for mPTP opening. In this study, we found that circBIRC6-2 plays a role in regulating mPTP opening by encoding BIRC6-236aa; therefore, we reasoned that BIRC6-236aa interacts with the VDAC1–CypD–ANT1 complex. Indeed, we found that BIRC6-236aa interacts with VDAC1 and attenuates the interaction between CypD and VDAC1 to inhibit opening of mPTPs, indicating that BIRC6-236aa and VDAC1 compete for CypD. This is a new pathogenic mechanism underlying mPTP opening.

In conclusion, we provide evidence that circBIRC6-2 encodes a protein, BIRC6-236aa, that suppresses TGEV-induced mPTP opening by competing with CypD for binding to VDAC1 (Fig. 8). These findings suggest that inhibiting TGEV-induced mPTP opening is a novel potential therapeutic approach to preventing TGEV-induced cell death.

## Experimental procedures

### Antibodies, cells, and virus

Anti-Flag rabbit monoclonal (14793) and anti-Tom20 rabbit monoclonal antibodies (mAbs) (42406) were purchased from Cell Signaling Technology. The anti-Flag mouse mAb was obtained from Sigma–Aldrich (F1804). The anti-HA mouse mAb was purchased from TransGen Biotech (HT301-01). The anti-HA rabbit mAbs (AB0025) and anti-CypD rabbit mAbs (CY7029) were purchased from Abways Technology. The anti-VDAC1 mAbs (rabbit and mouse; 55259-1-AP and 66345-1-Ig, respectively), the anti-ANT1 rabbit polyclonal antibody (15997-1-AP), and anti-SLC25A3 (15855-1-AP) were purchased from ProteinTech. The anti-β-actin mAb was purchased from Santa Cruz Biotechnology (sc-8432). The horseradish peroxidase–conjugated goat antimouse IgG (RK244131) and anti-rabbit IgG (RJ242536) were purchased from Thermo Fisher Scientific. Duolink PLA oligonucleotide-linked antimouse IgG (DUO92004) and anti-rabbit IgG (DUO92002) were purchased from Sigma–Aldrich. FITC-conjugated goat antimouse IgG (BA1101) and DyLight 594-conjugated goat anti-rabbit IgG (BA1142) were purchased from Boster. The IPEC-J2 cell line was a kind gift from Zhanyong Wei (Henan Agricultural University, China). IPEC-J2 cells were cultured at 37 °C/5% CO_2_ in Dulbecco's Modified Eagle’s Medium/F-12/HAM (Thermo Fisher Scientific), supplemented with 100 IU/ml penicillin and 100 μg/ml streptomycin. The TGEV Shaanxi strain was isolated from TGEV-infected piglets (54).

### Transmission electron microscopy

IPEC-J2 cells were collected in precooled fixation buffer (2.5% glutaraldehyde and 0.1 M phosphate buffer containing Na_2_HPO_4_ 12H_2_O and NaH_2_PO_4_ 2H_2_O) at 4 °C for 5 h. The cells were incubated for 2 h in osmic acid buffer (1% osmium tetroxide and 0.1 M phosphate buffer at pH 7.2–7.4) and dehydrated through a graded series of ethanol solutions. Next, the cells were embedded in a graded series of London Resin (LR) White resins and were finally embedded in fresh 100% LR White resin at 55 °C for 48 h. Then, samples were cut on a Leica UC7 ultramicrotome and collected on formvar-coated grids. Finally, the sections were poststained for 15 min with uranyl acetate and observed under a transmission electron microscope (TECNAI G2 SPIRIT BIO, FEI).

### Measurement of mitochondrial Ca^2+^ concentration

Mitochondrial Ca^2+^ concentration was measured using Rhod-2-AM kit (GenMed Scientific). The lipid-soluble Rhod-2-AM is nonfluorescent and the membrane-permeable form of Rhod-2. It can enter cells *via* incubation and does not bind Ca^2+^. AM group facilitates cellular uptake and is removed by cellular esterases, resulting in intracellular accumulation of Rhod-2. Rhod-2-AM has a weak positive charge that diffuses Rhod-2-AM into the highly polarized mitochondria matrix through membrane potential–driven uptake. The esterase activity is much higher in the mitochondria matrix than that in the cytosol such that the majority of Rhod-2-AM can traverse the cytosol to be taken up and then trapped in the mitochondria matrix following esterase cleavage. Rhod-2-AM is readily hydrolyzed into a membrane-impermeable Rhod-2 by cellular endogenous esterases once it enters cells. Rhod-2 is nonfluorescent before Ca^2+^ binding. Once binding Ca^2+^, Ca-Rhod-2 becomes more fluorescent with increasing Ca^2+^ concentration (55). Therefore, fluorescence intensity of Ca-Rhod-2 represents Ca^2+^ concentration.

IPEC-J2 cells were cultured in a black 96-well plate and washed twice in PBS. Next, 100 μl of cleaning buffer, 100 μl of saturated buffer, and 100 μl of negative buffer were respectively added to the sample wells, the maximum background wells (saturated calcium concentration group), and the minimum background wells (no calcium group). Then, Rhod-2-AM was added to all wells to a final concentration of 5 μM and incubated at 37 °C for 30 min. Ca-Rhod-2 fluorescence intensity was measured at wavelength of 550 nm/590 nm with spectrofluorometer. Mitochondrial Ca^2+^ concentration and fluorescence intensity are related according to the equation:

$$\left[\text{C}{{\text{a}}^{2}}^{+}\right]\;=\;{\text{K}}_{\text{d}}\;\left[\left(\text{F }-\;{\text{F}}_{\text{min}}\right)/\left({\text{F}}_{\text{max}}\;-\text{ F}\right)\right]$$

F is the fluorescence of the indicator at experimental calcium concentration. F_min_ is the fluorescence in the absence of calcium, and F_max_ is the fluorescence of the indicator at saturated calcium concentration. The K_d_ for calcium indicators in cells may be affected by a number of factors including pH, protein concentration, ionic strength, temperature, and viscosity. Thus, calibration of the K_d_ is necessary for accurate measurement of intracellular calcium concentrations. K_d_ = 570 nmol.

To image Ca^2+^ fluorescence, IPEC-J2 cells were cultured in a 48-well plate and washed twice in PBS. Cells were stained using 200 nM Mito-Tracker Green (C1048, Beyotime) at 37 °C for 20 min and treated with Rhod-2-AM (final concentration of Rhod-2-AM = 5 μM) at 37 °C for 30 min. Images were captured using a laser scanning confocal microscope (Leica TCS SP8). Green fluorescence indicates mitochondria that are stained with Mito-Tracker Green, whereas red fluorescence indicates Ca^2+^ concentration that is stained with Rhod-2.

### Measurement of the MMP

The MMP was measured using a JC-1 kit (C2003S, Beyotime). JC-1 can be used as an indicator of MMP in a variety of cell types. Changes in the membrane potential are presumed to be associated with the opening of the mPTP, allowing passage of ions and small molecules. JC-1 are positively charged, causing them to accumulate in the electronegative interior of the mitochondrion. JC-1 exhibits potential-dependent accumulation in the mitochondria, indicated by a green fluorescence emission at (∼529 nm) for the monomeric form of JC-1, which shifts to red (∼590 nm) with a concentration-dependent formation of red fluorescent aggregates of JC-1. Consequently, mitochondrial depolarization is indicated by a decrease in the red/green fluorescence intensity ratio. Thus, the red/green fluorescence intensity ratio represents MMP and is positively correlated with MMP.

IPEC-J2 cells were cultured in a 48-well plate for 24 h and treated with JC-1 staining solution at 37 °C for 20 min. Images were captured using a laser scanning confocal microscope. To measure JC-1 fluorescence intensity, IPEC-J2 cells were cultured in 96-well plate and treated with JC-1 staining solution at 37 °C for 20 min. The cells were then centrifuged at 600*g* at 4 °C. Fluorescence intensity was measured using a plate reader (SynergyH1, BioTek).

### Measurement of mPTP opening

mPTP assay kit (C2009S, Beyotime) was used to measure the level of mPTP opening. Calcein-AM is a nonpolar dye that enters and accumulates in cytoplasm including mitochondrion in living cells. Calcein-AM is nonfluorescent and can be hydrolyzed by intracellular esterase into a green fluorescent calcein, which is membrane impermeable. Calcein can form a complex compound with cobalt ion (Co^2+^) and then become nonfluorescent. Cobalt chloride (CoCl_2_) was used as the quench agent. Normally, when mPTP is closed, CoCl_2_ cannot enter the mitochondrion and then mitochondrion presents green fluorescence. When mPTP is opening, CoCl_2_ can enter the cytoplasm through opening mPTP and forming a complex compound with calcein, co-calcein, leading to quench of the calcein green fluorescence in the mitochondrion. Therefore, the intensity of green fluorescence represents the level of mPTP opening. Ionomycin is calcium ion carrier and carries massive extracellular Ca^2+^ into the intracellular and mitochondrial matrix, contributing to the mPTP opening. So, ionomycin is used as positive control.

Briefly, IPEC-J2 cells were collected and treated for 15 min at 37 °C with 0.5 μl of the calcein-AM, followed by 5 μl of quenching agent for 15 min at 37 °C. Next, fluorescence intensity was measured in a flow cytometer (BD FACSAria III, BD). Calcein fluorescence is measured by flow cytometry with 10, 000 events. The excitation and emission wavelengths of calcein are 492 nm and 518 nm, respectively. Flow cytometry data files were spanned to “.fcs” file to analysis the result. The FCS files were analyzed by FlowJo V10.7.1 to quantify mean fluorescence intensity of calcein according to the studies (56, 57). The mean fluorescence intensity of calcein was obtained by averaging fluorescent intensities from all the pixels in the image(s) from each well that is the ratio of the total fluorescence intensity/cell counts. Fluorescence intensity correlates negatively with the level of mPTP opening.

### Cell viability assay

Cell viability was measured using CCK8 (Beyotime, catalog no: #C0037). Briefly, 3, 000 cells were seeded into each well of the 96-well plate. Ten microliter of CCK8 was added to each well, which contained 200 μl medium. After 1 h incubation, the optical densities at a wavelength of 450 nm were measured by plate reader (SynergyH1, BioTek).

### Extraction of gDNA extraction, total RNA, and qRT-PCR

gDNA was extracted from IPEC-J2 cells using a Universal Genomic DNA Kit (Cwbiotech). Total RNA was extracted from jejunal tissues and IPEC-J2 cells using TRIzol reagent and reverse transcribed into cDNA using M-MLV reverse transcriptase (Promega). Next, qRT-PCR was conducted in triplicate using a QuantStudio 6 Flex Real-Time PCR System (Life Technologies) and *PerfectStart* Green qPCR SuperMix (TransGen Biotech). Relative expression of mRNA was assessed by normalization to β-actin and analyzed using the 2^-ΔΔCt^ method (58). The sequences of the primers used for PCR are provided in Table S2.

### Prediction of the circBIRC6-2 IRES sequence and performance of the dual-luciferase reporter assay

The circBIRC6-2 sequence was divided into small but equal segments. IRESfinder (https://github.com/xiaofengsong/IRESfinder) was used to analyze the sequence of each segment (59). Two circBIRC6-2 sequences (1–174 bp and 590–763 bp) were identified as potential IRES and cloned into the dual-luciferase reporter vector psiCHECK2 to yield psiCHECK2-IRES1 and psiCHECK2-IRES2, respectively. The plasmids were transfected into IPEC-J2 cells using Lipofectamine 3000 and p3000. A dual-luciferase reporter assay (Promega) was used to detect firefly luciferase and Renilla luciferase activity at 48 h post-transfection.

### FISH

Cells were infected for 12 h with TGEV at a MOI = 1.0. The sequence of circBIRC6-2 probe is GACTG + TAGGATGTGACCT + TAAACGG, which spans circBIRC6-2 junction. The circBIRC6-2 probe was labeled with Cyanine 3 (CY3), and FISH assay was performed using a FISH kit (GenePharma). Nuclei were counterstained with 4, 6-diamidino-2-phenylindole (DAPI), and images were captured with a laser scanning confocal microscope.

### Plasmid construction and transfection of oligonucleotides or plasmids

CircBIRC6-2 shRNA and negative control shRNA were chemically synthesized by GenePharma. The target gene sequence of circBIRC6-2 shRNA is CCGTTTAAGGTCACATCCT. Plasmid pcircBIRC6-2 contains a donor sequence (GCCACTAACTCTCTAATTGTTTTTTTTTTCAG) and a receptor sequence (GTAAGTATAAAATTTTTAAGTGTATAA). The two sequences are responsible for cRNA splicing. The target gene sequence was flanked by the donor sequence and receptor sequence. Proteins involved in formation of cRNA recognize the donor and receptor sequences, resulting in formation of cRNA.

Recombinant plasmid pcircBIRC6-2—the circBIRC6-2 sequence was cloned into the pCD513B-1 vector. The front-circular frame (FCF) and back-circular frame (BCF) were added to the 5′ and 3′ ends, respectively, of the circBIRC6-2 sequence to guarantee production of cRNA. Recombinant plasmid pcircBIRC6-2-Flag—a Flag-tag sequence was inserted after the “ATG” initiation codon in pcircBIRC6-2. Recombinant plasmid pcircBIRC6-2-Flag-Mut—the initiation codon “ATG” of BIRC6-236aa was mutated to “ACG” in the pcircBIRC6-2-Flag vector; therefore, it does not translate a protein. Recombinant plasmid pFlag-BIRC6-236aa—this plasmid generates linear RNAs encoding BIRC6-236aa.

The VDAC1 gene was cloned into plasmid pCAGGS-HA. To enable formation of RNA circular, sequences for the FCF and BCF were cloned into a CMV-induced expression vector (pCDH-CMV-MCS-EF1-copGFP-T2A-Puro, also called pCD513B-1). The ATG of the GFP ORF of pCD513B-1 was mutated into ACG. Then, the DNA sequences of circBIRC6-2, circBIRC6-2-Flag, and circBIRC6-2-Flag-Mut were inserted between the FCF and the BCF. The ORF of circBIRC6-2 and the Flag sequences were inserted into pCD513B-1. The primers used are listed in Table S2. Plasmids were transfected into IPEC-J2 cells using Lipofectamine 3000 or p3000. The final concentrations of siRNA and plasmid were 200 pmol/ml and 1 μg/ml, respectively.

### Nuclear-cytoplasmic fractionation and treatment of total RNA with RNase R

Nuclear and cytoplasmic RNA were separated using Cytoplasmic & Nuclear RNA Purification Kit (Norgen Biotek).

Briefly, total RNA was incubated with RNase R at 37 °C for 30 min and then transcribed into cDNA using M-MLV reverse transcriptase. The amount of RNA encoding circBIRC6-2, BIRC6, and β-actin was analyzed by qRT-PCR.

### Western blot analysis

IPEC-J2 cells were lysed for 30 min with radioimmunoprecipitation assay lysis buffer containing PMSF and then centrifuged at 12, 000*g* for 15 min. The proteins in the supernatant were separated by 12% SDS-PAGE and transferred to a polyvinylidene fluoride membrane (Millipore). The membrane was blocked at room temperature (RT) for 2 h with 5% nonfat milk and then incubated at 4 °C for 8 h with primary antibodies, followed by incubation with a secondary antibody at RT for 2 h. Signals were detected by enhanced chemiluminescence (Promega).

### Co-IP

IPEC-J2 cells were transfected with plasmids and subsequently infected with TGEV (1 MOI) at 12 hpi (hours post-infection). Then, cells were lysed in IP lysis buffer (Beyotime) and centrifuged at 12, 000*g*. The supernatant was incubated at 4 °C for 1 h with 50 μl of protein A/G agarose (Sigma–Aldrich) and then subjected to co-IP overnight at 4 °C using anti-Flag or anti-IgG. The protein complexes were incubated with 50 μl of protein A/G agarose at RT for 2 h and then analyzed by Western blotting.

### Analysis of peptide patterns by LC-MS/MS

The detailed experimental procedure has been described previously (60).

#### Protein isolation

Total proteins pulled down by IP were isolated by SDS-PAGE. The gels containing protein bands were collected and treated with a buffer containing 10 mM Tris (2-carboxyethyl) phosphine hydrochloride, 100% acetonitrile, 60 mM iodoacetamide, and 50 mM NH_4_HCO_3_. The proteins were then digested into peptides using trypsin buffer (2 μg trypsin and 50 mM NH_4_HCO_3_). The peptides were purified using Pierce C18 Tips (87784, Thermo Fisher Scientific).

#### LC-MS/MS

Peptides were fractionated using high pH reverse phase HPLC, separated on a reverse phase analytical column (Acclaim PepMap RSLC; Thermo Fisher Scientific), and analyzed using a Q ExactiveTM Hybrid Quadrupole-Orbitrap Mass Spectrometer (Thermo Fisher Scientific). The peptides were subjected to a nanoSpray ionization source, followed by MS/MS using a Q ExactiveTM Hybrid Quadrupole-Orbitrap Mass Spectrometer (Thermo Fisher Scientific) coupled in line with the HPLC. Intact peptides were detected in the Orbitrap at a resolution of 70, 000 and selected for MS/MS using normalized collision energy setting as 32. Ion fragments were detected in the Orbitrap at a resolution of 17,500. A data-dependent procedure that alternates between one MS scan followed by 20 MS/MS scans was applied to the top 20 precursor ions above a threshold ion count of 2E4 in the MS survey scan, with dynamic exclusion set at 30 s. The electrospray voltage was 2.0 kV. Automatic gain control was used to prevent overfilling of the ion trap. 5E4 ions were accumulated to generate MS/MS spectra. For the MS scans, the m/z scan ranged from 350 to 1800. Fixed first mass was set as 100 m/z.

#### Analysis of LC-MS/MS data

Briefly, raw MS data were processed using the LC/MS software Proteome Discoverer (version 2.2) (Thermo Fisher scientific) and converted into Proteome Discoverer generic format (mgf) files. Proteome Discoverer software was used for peak generation, precursor mass recalibration, extraction of the reporter ion intensity, and calculation of reporter ion intensity ratios. For each MS/MS spectrum, the ten most intense peaks in every 100 Da window were extracted for the database search. Then, a Proteome Discoverer search was performed by searching tandem mass spectra against Uniprot Sus Scrofa Database (UniProt release: April 10, 2018, 328,615 entries) (http://www.uniprot.org) concatenated with a reverse decoy database and the protein sequences of common contaminants. The cleavage enzyme was specified as trypsin/P. The maximum number of missing cleavages was set to 2. The mass tolerance for precursor ions was set to 10 ppm and that for MS/MS ions was set to 0.02 Da. Carbamidomethylation on Cys residues was specified as a fixed modification, as was oxidation on Met residues. Acetylation at the protein N terminus was specified as a variable modification. False discovery rate thresholds for proteins, peptides, and modified sites were specified at 1%. Proteome Discoverer software was used to assemble the peptide/protein groups, calculate the false discovery rate, and filter the identified proteins. Protein quantification was set to use only unique peptides bearing any modification. The median ratio of the reporter intensity of unique peptides was set as the relative change in protein abundance. Student’s *t* test was used to calculate the significance of the differences in relative protein abundance. A *p*-value < 0.05 was considered significant.

### IEM assay

BIRC6-236aa was overexpressed in IPEC-J2 cells. Next, cells were embedded in LR White resin as described previously and sectioned on a Leica UC7 ultramicrotome. Sections were blocked for 10 min in PBS containing 0.1% bovine serum albumin and then incubated with an anti-Flag antibody for 1 h at RT, followed by a secondary antibody (10 nm protein A-gold, BOSTER, GA1014, Wuhan, China) at RT for 30 min. Finally, sections were poststained for 15 min with uranyl acetate and viewed by transmission electron microscope (TECNAI G2 SPIRIT BIO).

### Immunofluorescence staining

IPEC-J2 cells were fixed for 20 min in 4% paraformaldehyde and washed three times with PBS. Cells were permeabilized for 10 min at RT in 0.2% Triton X-100 in PBS. Next, cells were blocked for 30 min at 37 °C with 5% bovine serum albumin in PBS. Subsequently, cells were incubated with an anti-Flag antibody (1:200) for 12 h at 4 °C. After washing three times with PBS, cells were incubated for 30 min at RT with FITC-conjugated goat antimouse IgG (1:200). Then, cells were exposed to a Tom20 primary antibody and a DyLight 594-conjugated goat anti-rabbit IgG antibody as described before. Finally, cells were washed three times with PBS and treated with DAPI at RT for 15 min. Images were captured under a laser scanning confocal microscope.

### DuoLink PLA

The PLA was performed as described previously (36). Briefly, pCAGGS-Flag, pCAGGS-Flag-BIRC6-236aa, or pCAGGS-CypD was transfected into IPEC-J2 cells. Cells were then infected for 12 h with 1 MOI TGEV and fixed immediately with 4% paraformaldehyde for 15 min at RT. Cells were permeabilized with 0.01% Tween 20/PBS for 10 min at RT, incubated with a mixture of two primary antibodies (mouse anti-Flag and rabbit anti-VDAC1) followed by an oligonucleotide-linked secondary antibody, and then exposed to polymerase and nucleotides (DUO92007, Sigma–Aldrich). Nuclei were counterstained with DAPI. Images were captured under a laser scanning confocal microscope.

### Animal experiments

Six newborn piglets were randomly assigned to two groups: Mock and TGEV. The Mock group was orally infected with 5 ml of PBS per piglet, whereas the TGEV group was orally infected with 5 ml of TGEV (10^7^ tissue culture infective dose 50 (TCID50)/ml). All piglets were euthanized at 48 h after onset of diarrhea, and the small intestine was collected immediately for RNA.

All animal experiment procedures were conducted in accordance with the guidelines of the Animal Ethics Committee of Northwest A&F University under document 2011-31101684. All experiments complied with the “Guidelines on Ethical Treatment of Experimental Animals” (2006), Number 398, set by the Ministry of Science and Technology, China, and had prior approval from the Experimental Animal Management Committee of Northwest A&F University (Approval ID: 20160612).

### Statistical analysis

Data are presented as the mean ± SD of three independent experiments. Statistical significance was analyzed using an unpaired Student′s *t* test. *p*-values of <0.05 (marked as ∗) and <0.01 (marked as ∗∗) were deemed significant and highly significant, respectively.

## Data availability

Data related to mass spectrometry, proteomics analysis, and single-peptide identification of BIRC6-236aa have been deposited to the ProteomeXchange Consortium *via* the PRIDE (61) partner repository under the dataset identifier PXD029055 (http://www.ebi.ac.uk/pride).

## Supporting Information

This article contains supporting information.

## Conflict of interest

The authors declare that they have no conflicts of interest with the contents of this article.
