# Supplementary figures and images for: A new circular RNA–encoded protein BIRC6-236aa inhibits transmissible gastroenteritis virus (TGEV)–induced mitochondrial dysfunction
Source: J Biol Chem. 2022 Jul 19;298(9):102280. doi: 10.1016/j.jbc.2022.102280 (PMC9400091; doi:10.1016/j.jbc.2022.102280)

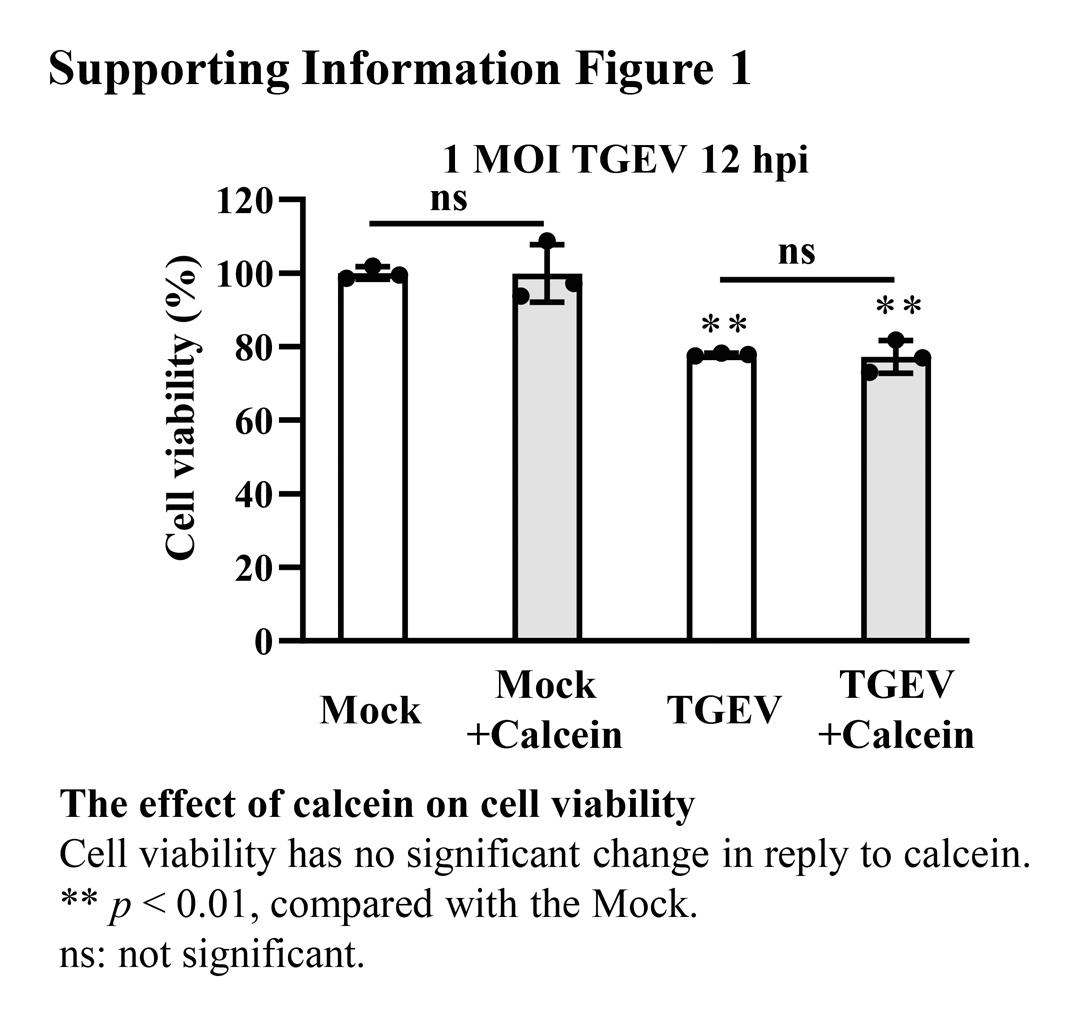

Supplement: Supporting Information Figure 1 [file figs1.jpg]

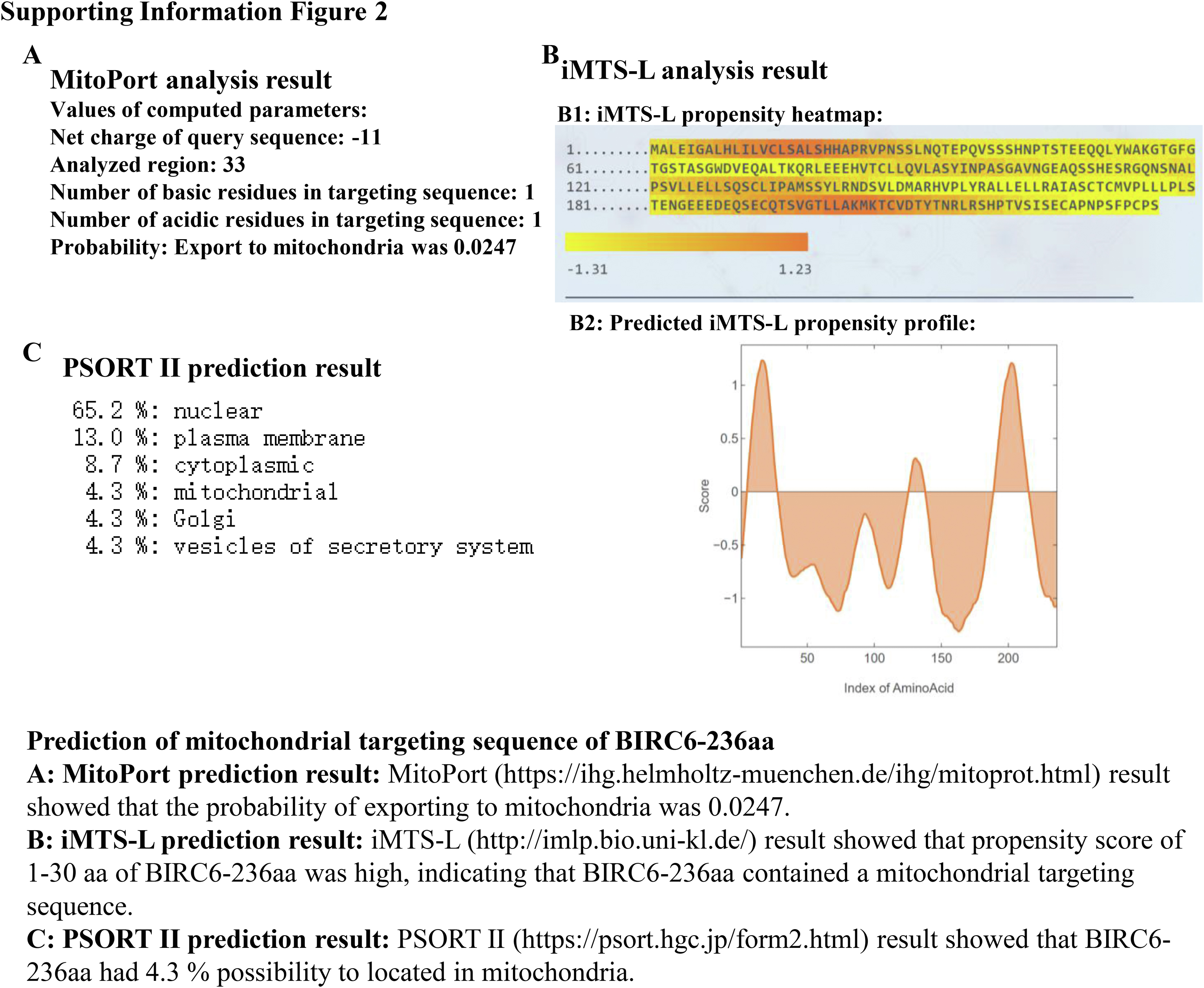

Supplement: Supporting Information Figure 2 [file figs2.jpg]

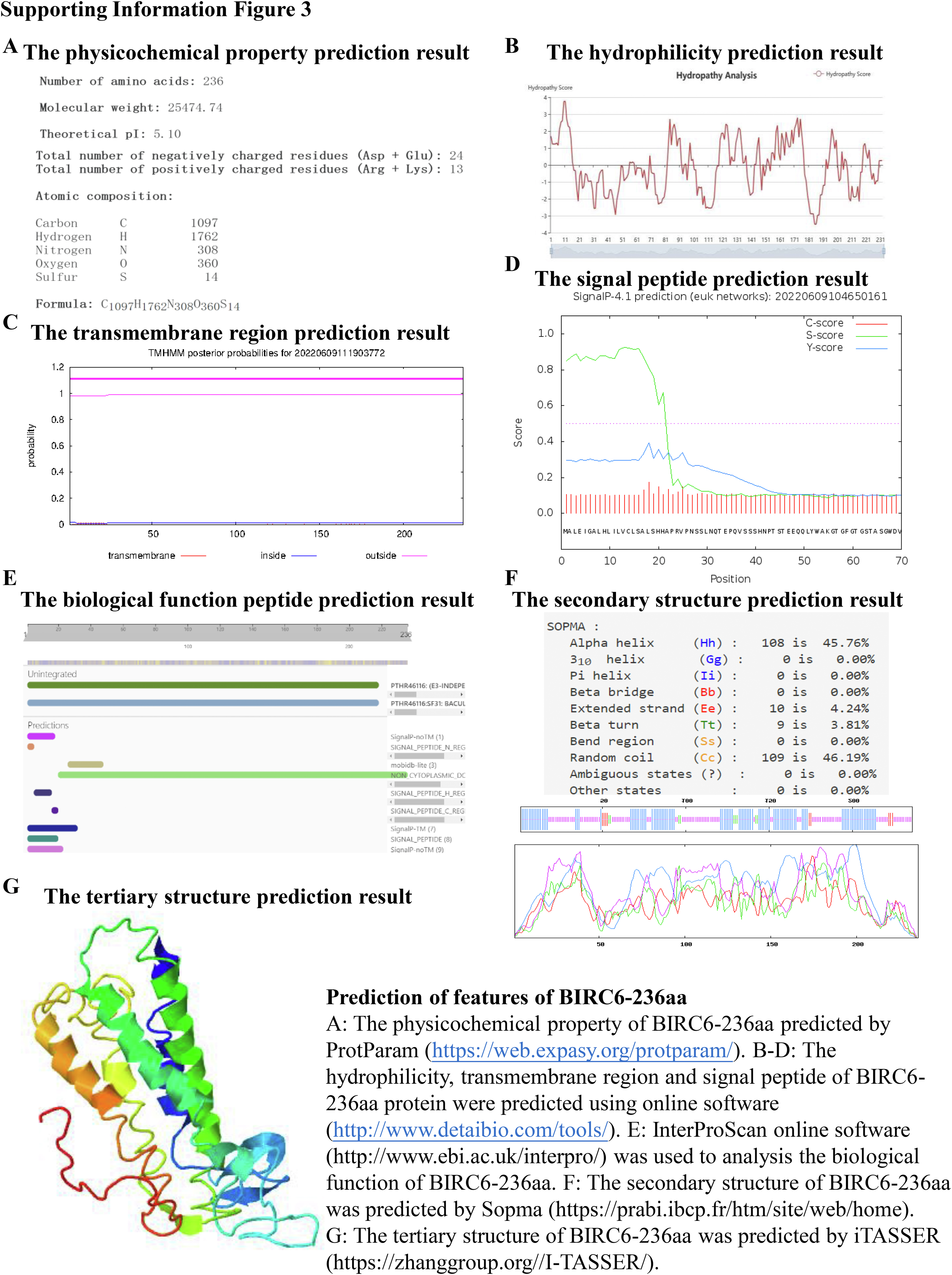

Supplement: Supporting Information Figure 3 [file figs3.jpg]

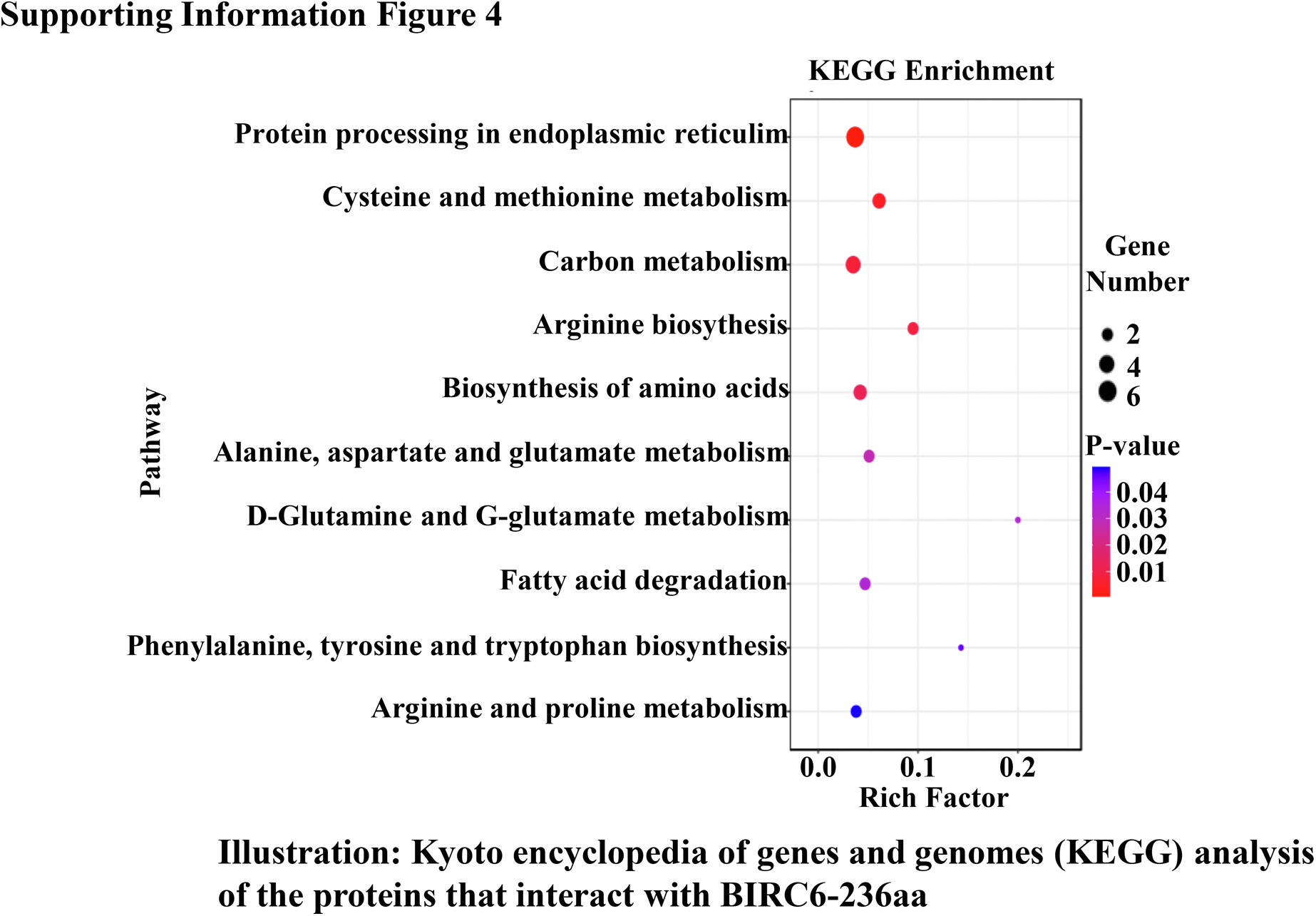

Supplement: Supporting Information Figure 4 [file figs4.jpg]

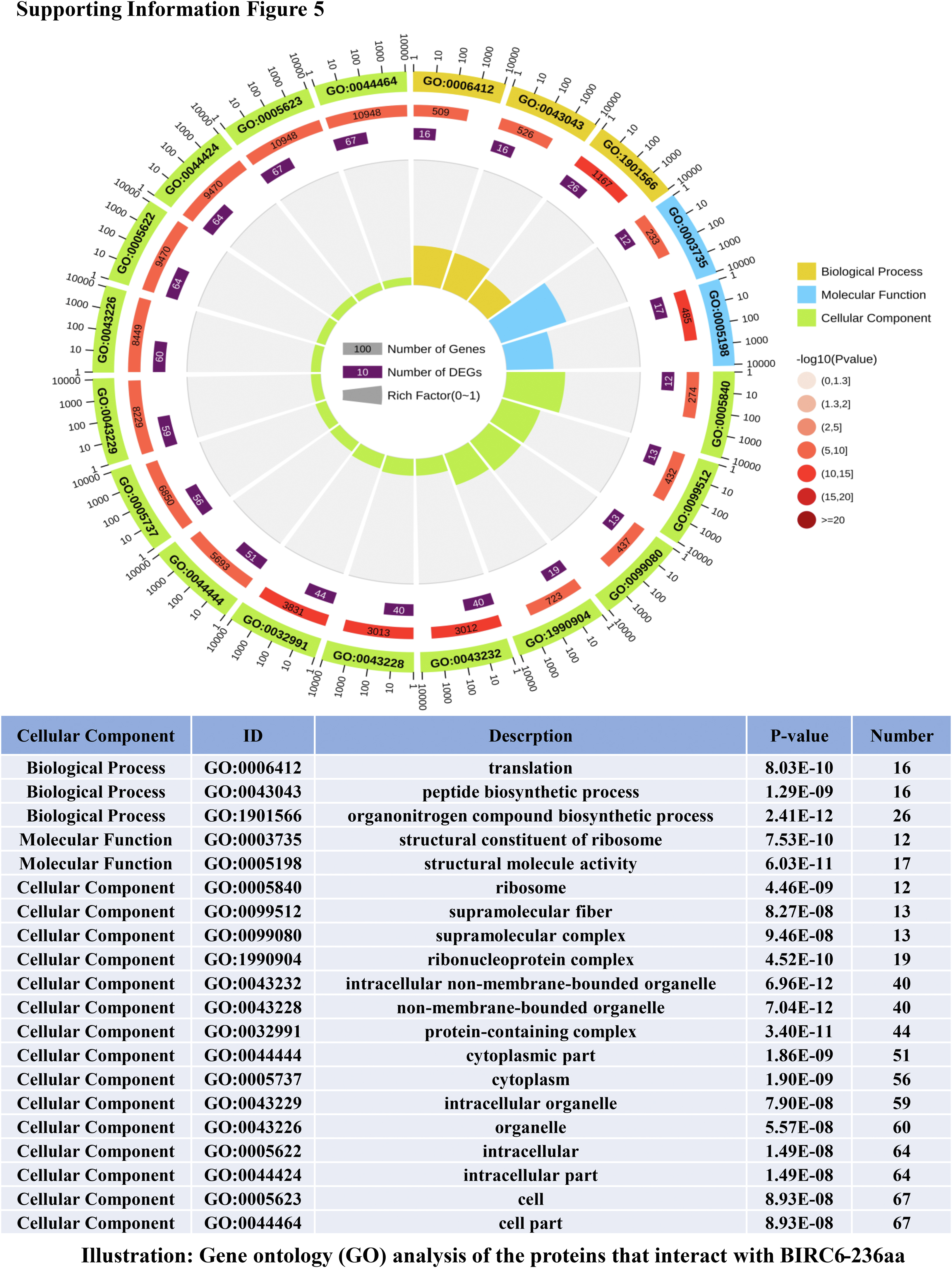

Supplement: Supporting Information Figure 5 [file figs5.jpg]
